# *Candida albicans* activates *Staphylococcus aureus* virulence regulatory systems to drive toxin-mediated human cell death

**DOI:** 10.1371/journal.ppat.1014490

**Published:** 2026-08-05

**Authors:** Kara R. Eichelberger, Ravishankar Chandrasekaran, Nicholas A. Podar, Jia A. Mei, Jacob M. Curry, Saikat Paul, Julie A. Bastarache, Victor J. Torres, Paul L. Fidel, Brian M. Peters, James E. Cassat

**Affiliations:** 1 Department of Pediatrics, Division of Pediatric Infectious Diseases, Vanderbilt University Medical Center, Nashville, Tennessee, United States of America; 2 Department of Host-Microbe Interactions, St. Jude Children’s Research Hospital, Memphis, Tennessee, United States of America; 3 Biomedical Engineering Program, School of Engineering, Vanderbilt University, Nashville, Tennessee, United States of America; 4 Department of Pathology, Microbiology, and Immunology, Vanderbilt University Medical Center, Nashville, Tennessee, United States of America; 5 Department of Clinical Pharmacy and Translational Science, University of Tennessee Health Science Center, Memphis, Tennessee, United States of America; 6 Department of Medicine, Division of Allergy, Pulmonary, and Critical Care Medicine, Vanderbilt University Medical Center, Nashville, Tennessee, United States of America; 7 Department of Oral and Craniofacial Biology, Louisiana State University Health – School of Dentistry, New Orleans, Louisiana, United States of America; 8 Department of Microbiology, Immunology, and Biochemistry, University of Tennessee Health Science Center, Memphis, Tennessee, United States of America; 9 Department of Biomedical Engineering, Vanderbilt University, Nashville, Tennessee, United States of America; 10 Vanderbilt Center for Bone Biology, Vanderbilt University Medical Center, Nashville, Tennessee, United States of America; 11 Vanderbilt Institute for Infection, Immunology, and Inflammation (VI4), Vanderbilt University Medical Center, Nashville, Tennessee, United States of America; National Institutes of Health, UNITED STATES OF AMERICA

## Abstract

Co-infection with *Staphylococcus aureus* and *Candida albicans* leads to worsened disease severity compared to mono-microbial infection. Because our understanding of the mechanisms driving enhanced disease severity during co-infection is incomplete, we sought to evaluate how interactions with *C. albicans* regulate *S. aureus* virulence toward host cells. We determined that *C. albicans* enhances *S. aureus* cytotoxicity toward both murine and human monocytes. These data revealed that enhanced murine monocyte cell death requires the *S. aureus* Agr virulence regulatory system, and cell death is driven by α-type phenol soluble modulins and γ-hemolysin. Unexpectedly, upon testing human monocytes we discovered that *C. albicans* induces robust cytotoxicity of an *S. aureus agr* mutant (Δ*agr*), which is completely non-toxic in mono-culture and toward murine cells. Human neutrophils are also susceptible to this cytotoxicity. Using reporter strains and combinatorial mutants, we identified that co-culture activates the SaeRS regulatory system in *S. aureus,* and SaeRS is required for human-specific cytotoxicity. We further discovered that the SaeRS-regulated toxin Panton-Valentine Leukocidin (PVL) drives *S. aureus* Δ*agr* cytotoxicity following co-culture. We observed similar cytotoxicity phenotypes using clinical isolates of both *S. aureus* and *C. albicans*, demonstrating broad conservation of this interaction. Finally, using mice that express the human isoform of the PVL receptor, we found that *C. albicans* enhances virulence of *S. aureus* Δ*agr* during co-infection. Overall, this study determined that *C. albicans* enhances *S. aureus* toxin-mediated host cell death, and co-culture engages a major virulence regulatory system in a typically non-toxic *S. aureus* strain to induce potent human-selective cytotoxicity.

## Author summary

Polymicrobial infections are often associated with worsened disease outcomes relative to monomicrobial infections, such as longer hospital stays and increased patient mortality. Interactions between co-infecting microorganisms can lead to changes in virulence that contribute to this enhanced disease severity. However, our understanding is limited regarding the specific microbial mechanisms that alter pathogenesis. In this study, we investigate how interactions with the common co-colonizing and co-infecting fungus *Candida albicans* regulate the virulence of bacterial pathogen *Staphylococcus aureus*. We discovered that interactions with *C. albicans* lead to greater *S. aureus* toxin-mediated human cell death, and we defined the bacterial virulence regulators and toxins driving this cytotoxicity. These results expand on our knowledge of how polymicrobial interactions can regulate staphylococcal virulence to enhance host cell death, which may contribute to greater tissue damage and worsened outcomes during co-infection.

## Introduction

*Staphylococcus aureus* is a major human pathogen and a leading cause of multiple invasive infections, including infective endocarditis, osteomyelitis, bacteremia, and pneumonia [1,2]. Colonization is an important risk factor for infection. It is estimated that up to 30% of adults are stably colonized with *S. aureus* at skin and mucosal sites, such as the nares, gastrointestinal tract, axillae, and groin [3]. *S. aureus* is also commonly co-isolated from polymicrobial communities associated with chronic infections [4,5]. Interactions with other bacteria that are present at colonization and infection sites can play a major role in influencing *S. aureus* virulence and treatment susceptibility [6–11]. Fungi are also important constituents of polymicrobial communities that harbor *S. aureus*, but how fungi interact with *S. aureus* to modulate virulence is less well defined. In this study, we sought to determine how interactions with the common co-infecting and co-colonizing fungus, *Candida albicans*, regulate staphylococcal virulence.

The human fungal opportunistic pathogen *C. albicans* is frequently isolated from the same host sites as *S. aureus*. *C. albicans* is among the most frequently detected species of the mycobiome present in the gastrointestinal tract, the oral cavity, and the vaginal tract of healthy people, and it can also be found on the skin [12–15]. The overlap in colonization niches may lead to a greater risk of polymicrobial disease by *C. albicans* and *S. aureus*, especially in patients with co-morbidities or chronic conditions. For instance, *C. albicans* and *S. aureus* are among the two most commonly identified fungal and bacterial species in burn wounds and diabetic foot ulcers [4,16,17], mixed fungal-bacterial bloodstream infections [18], polymicrobial peritonitis [19], and sputum samples from people with cystic fibrosis [20,21]. *C. albicans* polymicrobial infections are associated with greater disease severity compared to infection with either bacteria or fungi alone [19,22,23]. Because co-infection is associated with enhanced disease severity, it is important to define mechanisms of fungal-bacterial interactions that regulate virulence.

Murine models of intraabdominal and oral co-infection recapitulate the increased disease severity observed for *C. albicans* and *S. aureus* polymicrobial infections in humans [24–26]. In a murine model of polymicrobial intraabdominal infection, *C. albicans* and *S. aureus* co-infection results in higher mortality compared to mono-infection with either organism. Increased mortality in this model is driven by greater activation of the *S. aureus* accessory gene regulator, or Agr, system [27,28]. Agr is a quorum-sensing two-component system that regulates the production of multiple virulence factors in *S. aureus*, including secreted cytolytic peptides and toxins [29,30]. Agr-regulated peptides include the α-type phenol soluble modulins (αPSMs), which exhibit receptor-dependent immunomodulatory effects and receptor-independent cytolytic properties toward host cells [31,32]. Additional secreted toxins regulated by Agr bind specific receptors on host cells, initiating pore formation that induces host cell lysis and contributes to tissue damage during infection [33]. The importance of Agr-regulated toxins to *S. aureus* pathogenesis and disease severity is underscored by the fact that *S. aureus agr* mutant strains are attenuated in several murine infection models [34–36]. Yet, *S. aureus* strains with *agr* mutation or reduced Agr activity are frequently isolated from several human invasive infections or chronic conditions, such as bacteremia, osteomyelitis, and from the lungs of people with cystic fibrosis [37–39]. Because many of these chronic infections are polymicrobial, *S. aureus* isolates with Agr dysfunction may still be influenced by interactions with other microbes, including *C. albicans*, at the infection site.

*S. aureus* is a multi-species pathogen, and several virulence factors (such as the Agr-regulated αPSMs) target host cells from multiple species, including humans and mice. Traditional murine models have been employed to great effect in untangling the roles of such toxins in *S. aureus* pathogenesis during infection [31,40]. However, *S. aureus* has evolved as a human-adapted pathogen, and humans were identified as a major hub of *S. aureus* transmission to other host species [41,42]. As a result of this adaptation, many *S. aureus* strains encode human-selective virulence factors. For instance, the toxins LukAB, LukSF-PV (PVL), and ɣ-hemolysins HlgCB, robustly bind the human isoform of their receptor but poorly bind the murine isoform [43–45]. Furthermore, *S. aureus* iron acquisition from hemoglobin and inhibition of innate immune cell chemotaxis is more potent toward human cells compared to murine cells [46,47]. Therefore, while murine systems are important for modeling disease, they can only partly recapitulate *S. aureus* pathogenesis. In this study, we investigated how interactions with *C. albicans* impact *S. aureus* virulence toward human and murine cells, with a focus on delineating how *S. aureus* cytotoxicity is enhanced via both Agr-dependent and Agr-independent mechanisms.

## Results

### *C. albicans* induces *S. aureus* Agr-dependent cytotoxicity toward murine monocytes

**Fig 1:**
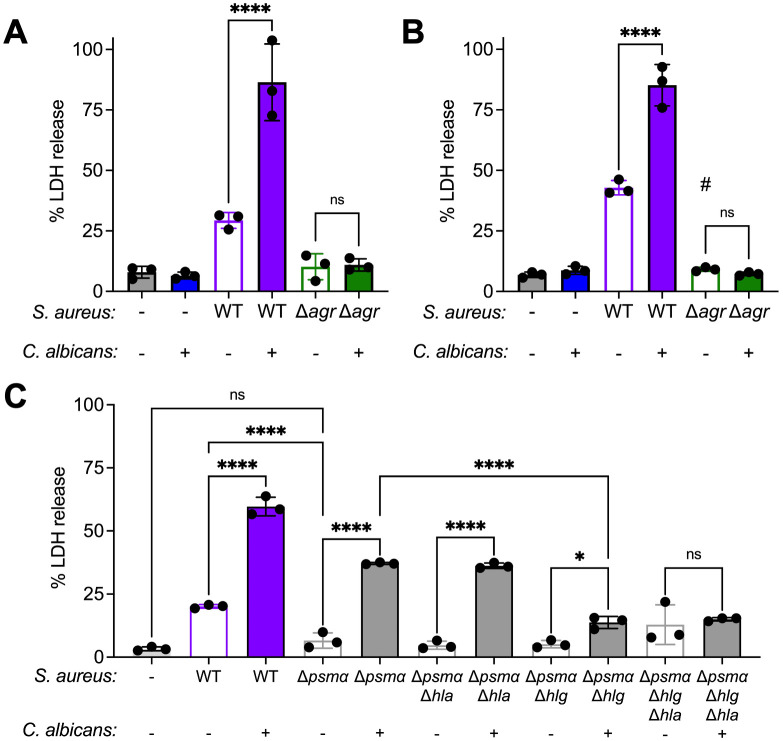
*C.* *albicans* enhances *S. aureus* cytotoxicity toward murine monocytes via Agr-regulated α-type phenol soluble modulins and γ-hemolysin. Murine bone marrow-derived monocytes were exposed to culture supernatants from *S. aureus* strains grown with or without *C. albicans.* % LDH release was quantified at (A) 6 hours and (B-C) 24 hours after supernatant exposure. *S. aureus* WT = wild type; Δ*agr* = *agrBDCA::tet*; Δ*psmα* = *psmα1–4*::*erm*; Δ*hla* = *hla*::*spc*; Δ*hlg* = *hlgACB::tet*. *p < 0.05, ****p < 0.001, and ns (not significant) by one-way ANOVA with Tukey’s multiple comparisons test. # represents p < 0.001 compared to *S. aureus* WT mono-culture*.* N = 3 wells per supernatant condition, error bars = mean ± SD.

Agr-regulated toxins are important for *S. aureus* to trigger immune cell death. Because *C. albicans* enhances *S. aureus* Agr activation during co-culture [27], we evaluated how *C. albicans* co-culture impacts *S. aureus* cytotoxicity toward monocytes, which are highly susceptible to multiple Agr-regulated toxins [48]. We cultured *S. aureus* USA300 LAC* wild type [49], and an isogenic *S. aureus agr* mutant (*agrBDCA::tet,* designated Δ*agr*) in mono-culture or in co-culture with *C. albicans* SC5314 wild type [50]. Murine bone marrow-derived monocytes (BMDM) were exposed to mono- and co-culture supernatants, and lactate dehydrogenase (LDH) release was quantified at 6 and 24 hours to determine monocyte cell death. When *S. aureus* wild type was co-cultured with *C. albicans,* it induced significantly more BMDM cell death (Fig 1A-1B). This enhanced cytotoxicity is not simply driven by greater microbial burdens in co-culture, because *S. aureus* and *C. albicans* CFU counts were similar in mono- and co-cultures (S1 Fig). *S. aureus* Δ*agr* was non-toxic toward BMDM, and *C. albicans* co-culture did not induce cytotoxicity (Fig 1A-1B). These results suggest that *C. albicans* enhances cytotoxicity of *S. aureus* via Agr-regulated factors, which is congruent with previously published work demonstrating that *C. albicans* enhances *S. aureus* Agr activation during co-culture [27].

We next sought to identify the Agr-regulated toxins driving the enhanced cytotoxicity observed during *S. aureus*-*C. albicans* co-culture. We first tested the role of the αPSMs because their expression is directly regulated by AgrA, and αPSMs contribute significantly to *S. aureus* cytotoxicity *in vitro* [29,51]. *S. aureus psmα1–4::erm* (designated as Δ*psmα*) mono-culture was non-toxic to BMDM, indicating that *S. aureus* wild type mono-culture cytotoxicity is primarily due to the cytolytic activity of αPSMs (Fig 1C). However, we observed that *C. albicans* induced significant cytotoxicity of *S. aureus* Δ*psmα,* suggesting that other cytolytic factors are involved in driving BMDM cell death following co-culture. We next tested if the Agr-regulated toxins α-toxin and γ-hemolysin AB (HlgAB) are required for enhanced co-culture cytotoxicity, because both toxins were previously shown to contribute to murine monocyte cell death [44,52]. *C. albicans* still induced cytotoxicity of *S. aureus psmα1–4::erm hla::spc* (designated Δ*psmα*Δ*hla*) to the same extent as *S. aureus* Δ*psmα* (Fig 1C). *S. aureus* psmα1–4::erm *hlgACB::tet* (designated Δ*psmα*Δ*hlg*) induced minimal BMDM cell death when grown in either mono- or co-culture, confirming a critical role for HlgAB in driving co-culture cytotoxicity toward BMDM (Fig 1C). Loss of γ-hemolysins and α-toxin alone did not impact *S. aureus* cytotoxicity (S2 Fig). Collectively, these data demonstrate that *C. albicans* enhances *S. aureus* cytotoxicity toward murine BMDM primarily through Agr-regulated production of αPSMs and γ-hemolysin.

### *C. albicans* induces cytotoxicity of *S. aureus* Δ*agr* toward human monocytes

**Fig 2:**
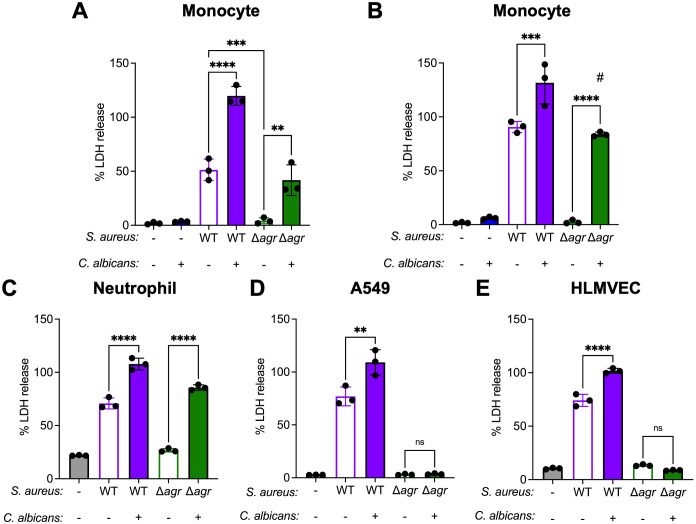
*C. albicans* induces *S. aureus* Δ*agr* cytotoxicity toward human monocytes and neutrophils. (A-B) Primary human CD14^+^ monocytes, (C) primary human neutrophils, (D) A549 human lung epithelial cells, and (E) human lung microvascular endothelial cells (HLMVEC) were exposed to culture supernatants from *S. aureus* cultured with or without *C. albicans.* % LDH release was quantified at (A) 6 hours and (B-E) 24 hours after supernatant exposure. *S. aureus* WT = wild type; Δ*agr* = *agrBDCA::tet*. **p < 0.01, ***p < 0.005, ****p < 0.001, and ns (not significant) by one-way ANOVA with Tukey’s multiple comparisons test. # represents ns compared to *S. aureus* WT mono-culture. N = 3 wells per supernatant condition, error bars = mean ± SD.

Because several *S. aureus* toxins selectively target human cells but not murine cells [33], we also tested if *C. albicans* induces *S. aureus* cytotoxicity toward primary human CD14^+^ monocytes. Co-culture induced significantly greater human monocyte cell death at both 6 and 24 hours after supernatant exposure (Fig 2A-2B). Cytotoxicity of *S. aureus* mono-culture is driven by Agr-regulated toxins, because *S. aureus* Δ*agr* was non-toxic toward human monocytes (Fig 2A-2B). Surprisingly, co-culture with *C. albicans* induced significant cytotoxicity of *S. aureus* Δ*agr* toward primary human monocytes as early as 6 hours after supernatant exposure (Fig 2A). By 24 hours, *S. aureus* Δ*agr* co-culture induced equivalent amounts of human monocyte death as *S. aureus* wild type mono-culture (Fig 2B). We confirmed that co-culture induced *S. aureus* Δ*agr-*mediated cell death in human monocytes using kinetic imaging with SYTOX Green (S3 Fig). These data reveal that in addition to enhancing Agr-dependent cytotoxicity, *C. albicans* is also able to induce *S. aureus* cytotoxicity independently of the Agr system, with specificity for human cells.

We next tested if *C. albicans* induces cytotoxicity of *S. aureus* Δ*agr* toward other human cell types. *S. aureus* wild type mono-culture triggered significant neutrophil cell death by 24 hours, and *C. albicans* co-culture enhanced this cytotoxicity (Fig 2C). *C. albicans* induced significant *S. aureus* Δ*agr* cytotoxicity toward human neutrophils, similar to what was observed with human monocytes (Fig 2C). To determine if *S. aureus* Δ*agr* is cytotoxic to non-immune cells following co-culture, we also tested human epithelial and endothelial cells. *C. albicans* significantly enhances *S. aureus* wild type cytotoxicity toward A549 human lung epithelial cells (Fig 2D) and human lung microvascular endothelial cells (HLMVEC) (Fig 2E). However, *C. albicans* failed to induce cytotoxicity of *S. aureus* Δ*agr* toward these cells*,* as both mono-culture and co-culture remained non-toxic (Fig 2D-2E). Taken together, these results demonstrate that *C. albicans* enhances both *S. aureus* Agr-dependent and Agr-independent cytotoxicity toward human cells, and co-culture induces cytotoxicity in a typically non-toxic strain of *S. aureus*. Additionally, human monocytes and neutrophils, but not epithelial and endothelial cells, are susceptible to Agr-independent cytotoxicity.

### *C. albicans* induces Agr-independent cytotoxicity toward human monocytes via *S. aureus* SaeRS

**Fig 3:**
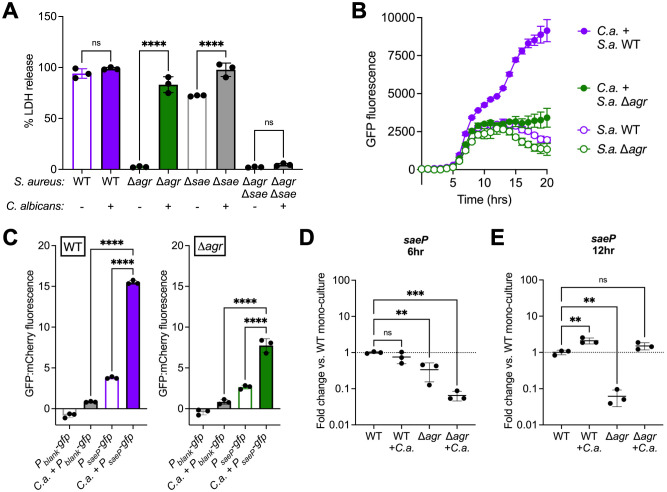
*C.* *albicans* co-culture induces *S. aureus* SaeRS activation, and SaeRS is required for induced *S. aureus* Δ*agr* cytotoxicity toward human monocytes. (A) Primary human CD14^+^ monocytes were exposed to supernatants from *S. aureus* strains cultured with and without *C. albicans*. % LDH release was quantified at 24 hours after supernatant exposure. (B-C) *S. aureus* wild type and Δ*agr P*_*saeP*_-*gfp* or *P*_*blank*_*-gfp* strains were cultured with and without *C. albicans,* and GFP and mCherry fluorescence values were measured every hour for 20 hours. (B) GFP fluorescence for each *P*_*saeP*_-*gfp* strain was blanked against the isogenic *P*_*blank*_*-gfp* control fluorescence values. (C) GFP fluorescence was standardized against mCherry fluorescence values for each strain in (B) at 15 hours. (D-E) qRT-PCR analysis of *saeP* transcript levels for cultures at 6 hours (D) and 12 hours (E) of culture. Values are plotted as fold change relative to the WT mono-culture samples. *C. albicans* = *C.a. S. aureus* WT = wild type; Δ*agr* = *agrBDCA::tet*; Δ*sae* = *saeQRS::spc*. For bar graphs, ****p < 0.001 by one-way ANOVA with Tukey’s multiple comparisons test; for qRT-PCR, **p < 0.01, ***p < 0.005, and ns (not significant) by one-way ANOVA with Dunnett’s multiple comparisons test. N = 3 wells or replicates per condition, bars or lines = mean ± SD.

Our discovery that *C. albicans* induces cytotoxicity of a *S. aureus agr* mutant strain toward human monocytes and neutrophils suggests that *C. albicans* increases Agr-independent production of human-specific toxins that target immune cells. While Agr is a major regulator of toxin production*,* the two-component system SaeRS also controls the production of multiple virulence factors including several toxins that demonstrate greater specificity for human cells compared to murine cells [53]. Because several studies demonstrate Agr-independent activity of SaeRS that induces toxin gene expression during *S. aureus* infection [39,54], we hypothesized that *C. albicans* induces *S. aureus* Δ*agr* cytotoxicity toward human cells via SaeRS. To test this, we first evaluated cytotoxicity of supernatants from *S. aureus* Δ*agr, saeQRS::spc* (designated Δ*sae*)*,* and an *agrBDCA::tet saeQRS::spc* double mutant (designated Δ*agr*Δ*sae*) cultured with and without *C. albicans*. *S. aureus* Δ*sae* mono-culture induced high levels of human cell death, and *C. albicans* co-culture significantly enhanced *S. aureus* Δ*sae* cytotoxicity toward human cells (Fig 3A). However, while *C. albicans* induced significant cytotoxicity of *S. aureus* Δ*agr,* co-culture failed to induce *S. aureus* Δ*agr*Δ*sae* cytotoxicity toward human monocytes (Fig 3A). These data support that *C. albicans* can enhance *S. aureus* cytotoxicity toward human cells through both Agr and SaeRS-regulated toxins. The results also suggest that *S. aureus* Δ*agr* co-culture cytotoxicity requires SaeRS.

To evaluate if co-culture activates *S. aureus* SaeRS-regulated gene expression, we used *S. aureus* wild type and Δ*agr* strains harboring either a chromosomally-integrated SaeRS-inducible promoter for *saeP* driving *gfp* expression (*P*_*saeP*_*-gfp*) or *gfp* without a promoter (*P*_*blank*_*-gfp*) as a control [55]. These reporter strains also expressed *mCherry* from a constitutive promoter (*P*_*sarA*_) [55]. *C. albicans* enhances *saeP* reporter activation relative to mono-culture for both *S. aureus* wild type and Δ*agr* (Fig 3B). The mCherry fluorescence values were similar among all strains and culture conditions tested (S4 Fig). Reporter activation for *saeP* was also significantly enhanced in co-culture relative to mono-culture when standardized against mCherry fluorescence levels at 15 hours (Fig 3C). Using qRT-PCR, we confirmed that *C. albicans* increased *saeP* transcript levels in co-culture for both *S. aureus* wild type and Δ*agr* by 12 hours (Fig 3D-3E). Based on this pattern of *saeP* gene expression, we tested cytotoxicity of *S. aureus* Δ*agr* mono- and co-culture after 6 and 12 hours of growth. The results show that 12-hour co-cultures induce significant human monocyte death (S5 Fig). This cytotoxicity correlates with when *saeP* transcript levels are increased in *S. aureus* Δ*agr* co-culture*.* Collectively, these data reveal that *C. albicans* induces *S. aureus* Δ*agr* cytotoxicity via a mechanism requiring SaeRS, and co-culture enhances SaeRS-regulated gene expression.

### *C. albicans* induction of *S. aureus* Δ*agr* cytotoxicity is mediated through Panton-Valentine Leukocidin (PVL)

**Fig 4:**
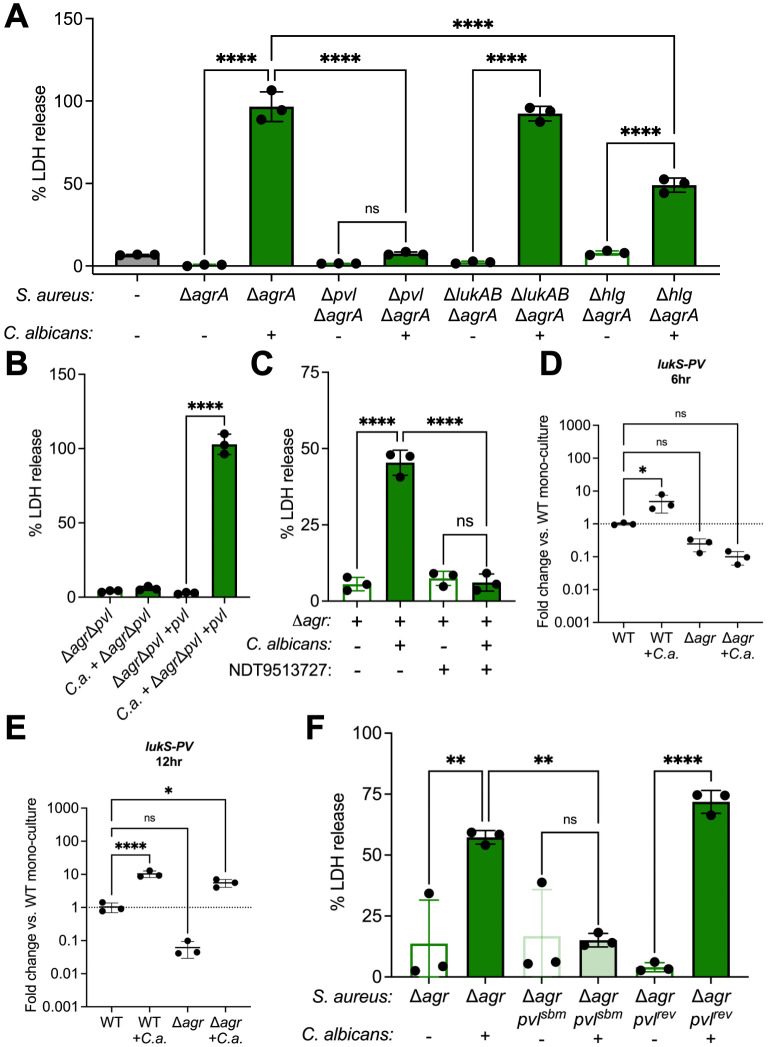
PVL is required for *S. aureus* Δ*agr* cytotoxicity induced by *C. albicans.* (A-B) Primary human CD14^+^ monocytes were exposed to supernatants from *S. aureus* strains cultured with and without *C. albicans*. % LDH release was quantified at 24 hours after supernatant exposure. (C) Monocytes were treated with 10 µM NDT9513727 or DMSO prior to the addition of culture supernatant and % LDH release was quantified at 6 hours. (D-E) qRT-PCR analysis of *lukS-PV* transcript levels for cultures at (D) 6 hours and (E) 12 hours of growth. Values are plotted as fold change relative to the WT mono-culture samples. (F) CD14^+^ monocyte cell death was quantified by % LDH release at 24 hours after supernatant exposure. *S. aureus* WT = wild type; Δ*agr* = *agrBDCA::tet;* Δ*agrA = agrA::bursa*; Δ*pvl = lukSF-PV::spc*; Δ*hlg = hlgACB::tet,* Δ*agr*Δ*pvl* + *pvl* = *agrBDCA::tet lukSF-PV::spc attC*::p*pvl-pvl;* Δ*agr/pvl*^*sbm*^ (*agrBDCA::tet* SaeR binding site mutant strain)*,* Δ*agr/pvl*^*rev*^ (*agrBDCA::tet* SaeR binding site revertant strain)*.* For bar graphs, ****p < 0.001, **p < 0.01, and ns (not significant) by one-way ANOVA with Tukey’s multiple comparisons test; for qRT-PCR, *p < 0.05, ****p < 0.001, and ns (not significant) by one-way ANOVA with Dunnett’s multiple comparisons test. N = 3 wells or replicates per condition, bars = mean ± SD.

The data demonstrate that co-culture with *C. albicans* induces *S. aureus* Δ*agr* cytotoxicity toward human monocytes but not murine monocytes, and this enhanced cytotoxicity requires *S. aureus* SaeRS. Therefore, we hypothesized that enhanced co-culture cytotoxicity is driven by SaeRS-regulated toxin(s) that exhibit selective activity toward human cells. Previous work from our group revealed that several *S. aureus* pore-forming toxins have reduced abundance in the exoproteome of *S. aureus* Δ*sae* compared to *S. aureus* wild type [51]. Of these, LukAB, LukSF-PV (PVL), and ɣ-hemolysins HlgCB induce potent human cell lysis and have minimal activity toward murine cells [43–45]. To test if *S. aureus* Δ*agr* co-culture requires one of these three toxins for human cell death, we examined the cytotoxicity of *S. aureus* double mutants for each toxin in an *agrA* mutant background. Like *S. aureus* Δ*agr*, *agrA::bursa* (designated Δ*agrA*) was non-toxic toward human monocytes when grown in mono-culture, but *C. albicans* induced significant cytotoxicity (Fig 4A). We found that co-culture still induced cytotoxicity of Δ*agrA* Δ*lukAB* and Δ*agrA hlgACB::tet* toward human monocytes (Fig 4A). In contrast, *C. albicans* failed to induce cytotoxicity of Δ*agrA lukSF-PV::spc* toward human monocytes (Fig 4A), demonstrating that PVL is the main toxin required for *S. aureus* Δ*agr* co-culture cytotoxicity*.* Mono-cultures of *S. aureus* Δ*lukAB, S. aureus lukSF-PV::spc,* and *S. aureus hlgACB::tet* all induced significant human monocyte death, supporting that multiple Agr- and Sae-regulated toxins contribute to *S. aureus* wild type cytotoxicity (S6 Fig).

To confirm the role of PVL in driving *S. aureus* Δ*agr* cytotoxicity during co-culture, we complemented *S. aureus agrBDCA::tet lukSF-PV::spc* (designated Δ*agr*Δ*pvl*) with *lukSF-PV* at a neutral site on the chromosome. Complementation successfully restored the ability of *C. albicans* to induce cytotoxicity of *S. aureus* Δ*agr*Δ*pvl* (Fig 4B). We next tested if pharmacological blockade of C5aR1, the PVL receptor, would inhibit monocyte cell death. Pre-treatment of human monocytes with NDT9513727, a C5aR1 negative allosteric modulator [56,57], was sufficient to inhibit Δ*agr* co-culture cytotoxicity (Fig 4C). Furthermore, qRT-PCR confirmed increased *lukS-PV* transcript levels in *S. aureus* wild type and Δ*agr* co-culture (Fig 4D-4E).

We next sought to determine if direct regulation of PVL by SaeR contributes to cytotoxicity induced by *C. albicans*. First, an SaeR binding site consensus sequence upstream of *lukSF-PV* was identified and mutated in *S. aureus* Δ*agr,* generating *S. aureus* Δ*agr/pvl*^*sbm*^*.* We complemented this strain by reverting the mutated SaeR binding site sequence back to wild type, generating *S. aureus* Δ*agr/pvl*^*rev*^. *C. albicans* induces minimal cytotoxicity of *S. aureus* Δ*agr/pvl*^*sbm*^ toward human monocytes, and cytotoxicity of *S. aureus* Δ*agr/pvl*^*rev*^ co-culture is restored to a similar level as *S. aureus* Δ*agr* co-culture (Fig 4F). Taken together, our data reveal that *C. albicans* enhances *lukS-PV* expression in *S. aureus* Δ*agr,* and PVL is required for *S. aureus* Δ*agr* cytotoxicity toward human monocytes following co-culture. Furthermore, *C. albicans* enhances PVL-mediated human cell death in part through direct SaeR regulation upstream of *lukSF-PV*.

### *C. albicans*-induced cytotoxicity is conserved across multiple S. aureus clinical isolates

**Fig 5:**
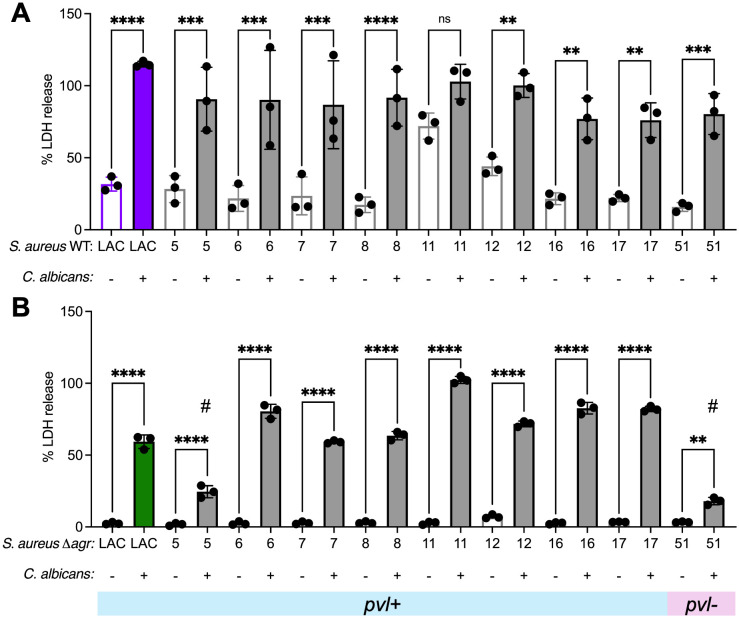
*C.* *albicans* co-culture enhances cytotoxicity of *S. aureus* USA300 clinical isolates and Δ*agr* strains. Primary human CD14^+^ monocytes were exposed to supernatants from (A) *S. aureus* LAC* and sequence type 8 clinical isolates and (B) the isogenic Δ*agr* mutant for each isolate, both cultured with and without *C. albicans*. % LDH release was quantified at (A) 6 hours or (B) 24 hours after supernatant exposure. All strains are *pvl+* (encode *lukSF-PV*) except for VUSA51. **p < 0.01, ***p < 0.005, ****p < 0.001, and ns (not significant) by one-way ANOVA with Tukey’s multiple comparisons test. # represents p < 0.001 compared to *S. aureus* LAC* Δ*agr* co-cultured with *C. albicans.* N = 3 wells or replicates per condition, bars = mean ± SD.

To determine if enhanced cytotoxicity following co-culture is conserved among *S. aureus* strains, we tested multiple *S. aureus* clinical isolates. We chose 9 *S. aureus* isolates that were classified as sequence type 8, which is the same sequence type as the LAC* wild type strain we use in this study [58]. Primary human monocytes were exposed to mono- and co-culture supernatants, and LDH release was evaluated at 6 hours. The results revealed that *C. albicans* significantly enhances cytotoxicity toward human monocytes for all of the strains tested except for isolate VUSA11, which induces high levels of cell death for both mono- and co-culture (Fig 5A). By 24 hours after supernatant exposure, all *S. aureus* wild type isolates cultured alone or with *C. albicans* induced maximal cell death (S7 Fig).

To evaluate if *C. albicans* induces cytotoxicity of *S. aureus* isolates with *agr* mutation, we generated Δ*agr* (*agrBDCA::tet*) strains for each of the clinical isolates in Fig 5A and tested cytotoxicity toward human monocytes. We confirmed that mutation of the Agr system rendered each *S. aureus* clinical isolate non-toxic when grown in mono-culture (Fig 5B). Furthermore, *C. albicans* induced cytotoxicity for 7 of the 9 clinical isolates to the same extent or greater than LAC* Δ*agr* co-culture (Fig 5B). We next performed whole genome sequencing for all *S. aureus* isolates to investigate why the isolates VUSA5 and VUSA51 were less cytotoxic following co-culture. Sequencing revealed that VUSA51 is PVL- while VUSA5 is PVL + . However, VUSA5 has a mutation that introduces an early stop codon in *arlS,* which is known to regulate *lukSF-PV* expression [59]. Collectively, these data suggest that *C. albicans* enhances cytotoxicity of multiple sequence type 8 clinical isolates and the isogenic Δ*agr* strains.

### *C. albicans* clinical isolates and mutant strains enhance *S. aureus* cytotoxicity

**Fig 6:**
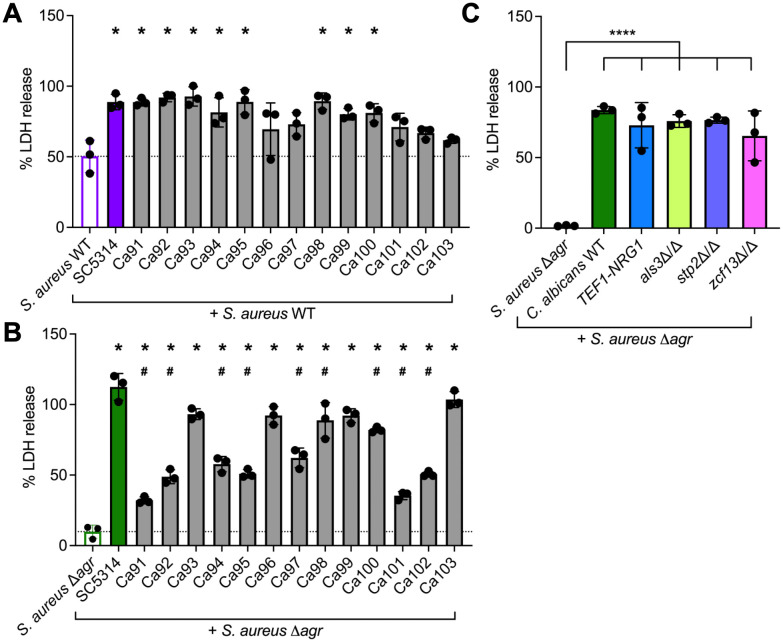
*C. albicans* clinical isolates and mutants enhance *S. aureus* cytotoxicity at variable levels. Primary human CD14^+^ monocytes were exposed to supernatants from *S. aureus* wild type (A) or *S. aureus* Δ*agr* that was cultured with and without *C. albicans* SC5314, the indicated *C. albicans* clinical isolates (B), or *C. albicans* mutants (C). % LDH release was quantified at 6 hours (A) or 24 hours (B-C) after supernatant exposure. *S. aureus* WT = wild type; Δ*agr* = *agrBDCA::tet*. Statistics were determined by one-way ANOVA with Tukey’s multiple comparisons test. * represents p < 0.01 and **** represents p < 0.001 compared to the mono-culture, and # represents p < 0.01 compared to *C. albicans* SC5314 co-culture. Dashed lines represent the amount of cytotoxicity induced by *S. aureus* mono-culture. N = 3 wells or replicates per condition, bars = mean ± SD.

High levels of phenotypic variation exist among *C. albicans* isolates. Therefore, we examined the ability of *C. albicans* clinical isolates to enhance *S. aureus* wild type and Δ*agr* cytotoxicity compared to *C. albicans* SC5314, the wild type reference strain used throughout this study. *S. aureus* wild type and Δ*agr* were cultured with *C. albicans* SC5314 or *C. albicans* clinical isolates. Because *C. albicans* co-culture can alter media pH and impact *S. aureus* virulence regulation [60], we first measured the pH of the co-cultures and confirmed that the *C. albicans* clinical isolates neutralized the media to a similar extent as *C. albicans* SC5314 (S8A-S8B Fig). We also confirmed that *S. aureus* growth was similar in co-culture with each clinical isolate (S8C-S8D Fig). Next, we observed that 8 of the *C. albicans* isolates tested significantly enhanced cytotoxicity of *S. aureus* wild type toward human monocytes, as determined by LDH release at 6 hours (Fig 6A). Each clinical isolate also induced cytotoxicity of *S. aureus* Δ*agr*, as measured by LDH release at 24 hours (Fig 6B). However, 9 of the isolates induced significantly less cytotoxicity relative to *C. albicans* SC5314 co-culture (Fig 6B). These data indicate that the ability to enhance *S. aureus* cytotoxicity is broadly conserved among *C. albicans* isolates, but the degree to which *C. albicans* isolates induce Δ*agr* cytotoxicity is more variable.

We next tested if previously described *C. albicans* mechanisms for enhancing *S. aureus* virulence are required for inducing *S. aureus* Δ*agr* cytotoxicity. *S. aureus* preferentially binds to *C. albicans* hyphae via the Als3p adhesin, and this binding promotes greater bacterial dissemination [61]. However, both a yeast-locked strain of *C. albicans* (*C. albicans TEF1-NRG1*) and *C. albicans als3*Δ/Δ significantly induced *S. aureus* Δ*agr* cytotoxicity (Fig 6C). *C. albicans* enhances *S. aureus* Agr activation via activities of the transcriptional regulators Stp2p and Zcf13p [28,60]. Therefore, we tested cytotoxicity of *C. albicans stp2*Δ/Δ and *zcf13*Δ/Δ co-cultured with *S. aureus* Δ*agr* and observed that these mutants both induce significant human cell death (Fig 6C). These results suggest that the *C. albicans* mechanism of enhanced Sae-mediated cytotoxicity differs from those previously described for physical interactions and Agr activation in *S. aureus.*

### *C. albicans* induces *S. aureus* Δ*agr* virulence in human C5aRI^KI^ mice

**Fig 7:**
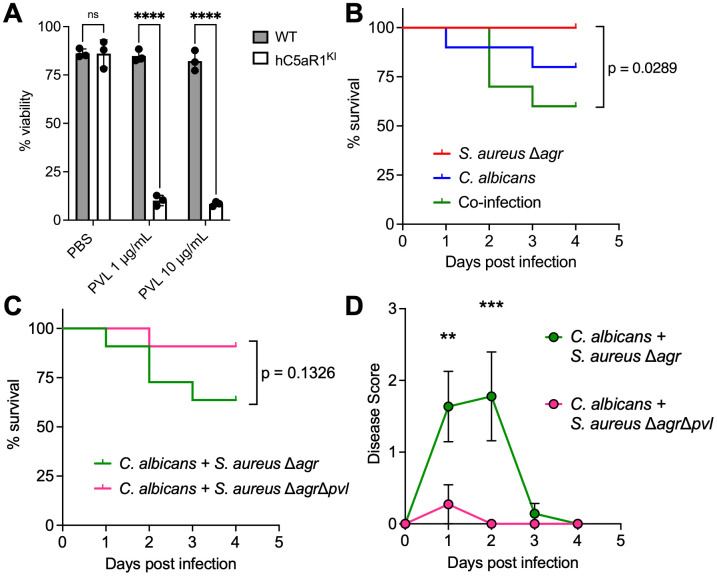
*C.* *albicans* enhances *S. aureus* Δ*agr* virulence in hC5aR1^KI^ mice. Survival of peritoneal exudate cells isolated from WT or hC5aR1^KI^ mice exposed to purified PVL toxin or PBS (A). % viability represents the proportion of eFluor660^-^ (live) CD45^+^CD11b^+^ cells. N = 3 wells or replicates per condition, bars = mean ± SD. Survival (B-C) and disease scoring (D) were determined for hC5aR1^KI^ mice (n = 10–11 per group) inoculated via intraperitoneal injection with *S. aureus* Δ*agr, C. albicans,* or *C. albicans* co-culture with either Δ*agr* or Δ*agr*Δ*pvl*. *S. aureus* Δ*agr* = *agrBDCA::tet;* Δ*pvl = lukSF-PV::spc*. Statistics in (A) and (D) were determined by two-way ANOVA with Šídák’s multiple comparisons test. **p < 0.01; ***p < 0.005; ****p < 0.001; ns = not significant. Significance in (B-C) were determined by Log-Rank (Mantel-Cox) test comparing Co-infection vs. *S. aureus* Δ*agr* (B) or *C. albicans* + *S. aureus* Δ*agr* vs. *C. albicans* + *S. aureus* Δ*agr*Δ*pvl* (C).

To test if *C. albicans* enhances *S. aureus* Δ*agr* virulence *in vivo* in a PVL-dependent manner, we used a mouse strain that expresses the human isoform of C5aR1 (hC5aRI^KI^) [62]. First, we tested the susceptibility of peritoneal exudate cells (PEC) of the myeloid lineage (CD45^+^CD11b^+^) to PVL-mediated cell death. We measured greater cytotoxicity for hC5aR1^KI^ cells exposed to purified PVL toxin, while cells isolated from wild type mice were not susceptible to PVL-mediated lysis (Fig 7A). Next, we inoculated hC5aRI^KI^ mice via intra-peritoneal injection. Inoculation of *C. albicans*-*S. aureus* Δ*agr* co-culture induced significantly greater mortality relative to mice inoculated with *S. aureus* Δ*agr* mono-culture (Fig 7B). Mice inoculated with *C. albicans* mono-culture growth had an intermediate survival phenotype relative to both groups (Fig 7B). To test if PVL is required for the enhanced mortality during *C. albicans-S. aureus* Δ*agr* co-infection, we inoculated hC5aRI^KI^ mice with *C. albicans-S. aureus* Δ*agr*Δ*pvl* co-culture*. C. albicans-S. aureus* Δ*agr* co-culture induced greater mortality compared to mice inoculated with *C. albicans-S. aureus* Δ*agr*Δ*pvl,* although this was not statistically significant (Fig 7C). Morbidity scoring was performed for each mouse in Fig 7C, and we observed significantly higher morbidity scores at 1 and 2 days post inoculation in the *C. albicans-S. aureus* Δ*agr* infection group compared to the *C. albicans-S. aureus* Δ*agr*Δ*pvl* infection group (Fig 7D)*.* Collectively, these data suggest that *C. albicans* enhances *S. aureus* Δ*agr* virulence *in vivo* in a PVL-dependent manner.

## Discussion

Using both human and murine cells, we determined that *C. albicans* enhances *S. aureus* virulence by engaging two staphylococcal virulence regulatory systems to induce cell death with host species-specific effects. While *C. albicans* enhances *S. aureus* cytotoxicity toward murine monocytes through the activity of Agr-regulated toxins, we uncovered that *C. albicans* unexpectedly induces cytotoxicity of the typically non-toxic *S. aureus agr* mutant, with selectivity toward human monocytes and neutrophils. Our data reveal that *C. albicans* can induce *S. aureus* SaeRS-mediated gene expression independently of Agr, and SaeRS is required for cytotoxicity toward human cells. We further identified PVL as the main SaeRS-regulated toxin contributing to human monocyte cell death when *S. aureus* Δ*agr* is co-cultured with *C. albicans.* Using mice that express the human isoform of the PVL receptor, we determined that *C. albicans* co-infection enhances *S. aureus* Δ*agr* virulence. These data expand our understanding of mechanisms by which fungal-bacterial interactions regulate staphylococcal virulence.

The *S. aureus* Agr system is regarded as a major virulence regulatory system due in part to the large repertoire of secreted toxins whose production is increased following Agr activation [30,33]. Previous work revealed that *C. albicans* enhances *S. aureus* Agr activation to elaborate α-toxin production, contributing to greater mortality in a murine model of intraabdominal co-infection [27,28,60]. Our results build on this finding by demonstrating that *C. albicans* co-culture enhances *S. aureus* cytotoxicity toward murine monocytes via an Agr-dependent mechanism requiring the α-type PSMs and the leukocidin γ-hemolysin. We did not observe a significant role for α-toxin in mediating enhanced cytotoxicity toward monocytes, but this toxin likely plays a role in regulating enhanced cytotoxicity toward other murine cell types. Additional potential impacts of α-toxin (or other toxins) on hemostasis, coagulopathy, and organ damage may underlie *hla-*dependent phenotypes observed during intra-abdominal co-infection [27,63–65]. Alternatively, given the established impacts of metabolism on shaping interactions between *C. albicans* and *S. aureus,* differences in culture systems and available nutrients could also underlie differences in specific toxins enhanced during co-culture.

We also discovered that *C. albicans* enhances *S. aureus* virulence independently of the Agr system via a mechanism requiring SaeRS. Because inactivation of Agr reduces *sae* transcript levels, the SaeRS two-component system was initially thought to be activated by Agr [66]. In keeping with this framework for regulation, we observed reduced *saeP* transcript levels in the *S. aureus* Δ*agr* strain compared to wild type. This may also explain the result that while *C. albicans* co-culture significantly enhances *S. aureus saeP* reporter activation in *S. aureus* Δ*agr* relative to mono-culture, the overall activation is about 3-fold lower compared to levels for *S. aureus* wild type*.* However, it is important to note that *sae* transcript levels do not always correlate with the level of SaeR activity [67]. While we observe an Agr-dependent effect on *saeP* transcript levels, our data also support a significant role for SaeRS in driving human monocyte cytotoxicity independently of *S. aureus* Agr following *C. albicans* co-culture*.* Other studies have also demonstrated a role for SaeRS in promoting *S. aureus* virulence independently of the Agr system [39,54]. Thus, while some SaeRS activity may be enhanced downstream of Agr, SaeRS can also be activated by *C. albicans* independently of the Agr system. A limitation to this conclusion is that while the results demonstrate that *C. albicans* induces SaeR-regulated gene expression, we have not quantified SaeRS two-component system activation in the strictest sense*.* Future experiments will evaluate if SaeR phosphorylation by SaeS is increased in the presence of *C. albicans*.

Our findings suggest a potential clinical scenario where *S. aureus* strains that have lost or reduced Agr function may still be susceptible to virulence regulation by *C. albicans.* Agr dysfunction is associated with *S. aureus* isolates from chronic conditions or infections, including cystic fibrosis [68,69], osteomyelitis [38], and wounds [70]. Despite lower toxicity *in vitro,* isolates with reduced Agr activity are still capable of causing disease [71–74]. Colonizing strains of *S. aureus* also exhibit Agr dysfunction, and co-colonizing microorganisms can inhibit Agr activity through heterologous autoinducing peptides [6,8,75]. Specific studies on the prevalence of *Candida* at sites of *S. aureus agr* null isolates are lacking, as *Candida* is often classified as a contaminant or a harmless commensal and excluded from study [72,76,77]. However, many of the sites and diseases that have a higher propensity for *S. aureus* Agr dysfunction also have an increased rate of polymicrobial etiology. *Candida* is among the most common fungus isolated from infections and niches where *S. aureus agr* dysfunctional isolates are found, including polymicrobial bloodstream infections [18,78], wounds [17], biofilm-associated infections [79], and the oral cavity and airways [80,81]. Therefore, *Candida* present at these sites may enhance *S. aureus* virulence via activation of SaeRS in isolates that have reduced or inhibited Agr function, potentially enhancing disease severity.

We determined that the SaeRS-regulated leukocidin PVL was required for the enhanced cytotoxicity of *S. aureus* Δ*agr* following *C. albicans* co-culture. PVL is species-specific, with high levels of activity toward human and rabbit cells, but poor activity toward murine cells [43]. This necessitated the use of a transgenic mouse strain that expresses the human isoform of the PVL receptor, C5aR1, to test if *C. albicans* enhances *S. aureus* Δ*agr* virulence in a PVL-dependent manner *in vivo*. Co-infection induces significantly greater mortality relative to *S. aureus* Δ*agr* mono-infection. While the difference in mortality for hC5aR1^KI^ mice inoculated with *S. aureus* Δ*agr* co-culture compared to mice inoculated with *S. aureus* Δ*agr*Δ*pvl* co-culture is not statistically significant, there is a significant difference in disease scoring, suggesting a role for *C. albicans* induction of PVL in driving enhanced virulence of *S. aureus* Δ*agr in vivo*. The low level of mortality observed in mice inoculated with *S. aureus* Δ*agr*Δ*pvl* co-culture may be due to *C. albicans,* which induces some mortality on its own. Previous work found that PVL had a limited impact on *S. aureus* virulence in the hC5aR1^KI^ mice due to lack of the human CD45 co-receptor [62]. While we do see enhanced virulence during co-infection in the hC5aR1^KI^ mice, future experiments using dually transgenic mice that express human isoforms of both C5aR1 and CD45 may reveal even greater impacts of PVL in this model.

Through testing if *C. albicans* broadly enhances cytotoxicity of *S. aureus* clinical isolates with a transduced *agr* mutation, we found that two isolates (VUSA5 and VUSA51) had significantly lower cytotoxicity following co-culture*.* Of note, VUSA51 is PVL- and VUSA5 is PVL + , but VUSA5 harbors a point mutation in *arlS,* which introduces an early stop codon. ArlRS is a two-component system that positively regulates *lukSF-PV* expression [59]. Therefore, it is possible that VUSA5 is unable to fully produce PVL due to the mutation in *arlS*. These results highlight the importance of PVL in driving enhanced virulence of *S. aureus agr* mutant strains toward human monocytes and demonstrate the ability of *C. albicans* to enhance the virulence of *S. aureus* strains with Agr dysfunction, which may otherwise be considered less pathogenic*.* The rates of PVL+ strains have been decreasing since the peak of *S. aureus* USA300 infections in the 2000s and 2010s, but there are still a significant number of PVL+ strains isolated worldwide, although specific rates in each country vary widely [82]. For example, a recent study of invasive *S. aureus* isolates obtained from patients at a single hospital in Nashville, TN, USA determined that approximately 60% of isolates from 2010-2012 were PVL+ and 35% of isolates since 2014 are PVL+ [83].

The mechanism by which *C. albicans* induces *S. aureus* SaeRS activation during co-culture is unknown. Several factors have been shown to activate SaeRS in *S. aureus* grown alone, although the precise mechanism of action is incompletely understood. SaeRS transcriptional activity was previously shown to be higher when *S. aureus* is grown in neutral pH compared to acidic pH [84]. We identified several *C. albicans* clinical isolates that induce significantly less Δ*agr* cytotoxicity compared to *C. albicans* SC5314, but these strains are still able to maintain neutral pH during co-culture. This indicates that neutral pH alone is not sufficient to permit SaeRS-mediated cytotoxicity during co-culture. Additionally, several *C. albicans* isolates that were poor inducers of *S. aureus* Δ*agr* cytotoxicity could significantly enhance cytotoxicity of *S. aureus* wild type. We further determined that *C. albicans STP2* and *ZCF13,* which are required for enhanced *S. aureus* Agr activation, are not required for *S. aureus* Δ*agr* cytotoxicity. These results suggest that *C. albicans* may engage *S. aureus* Agr and Sae systems via distinct mechanisms, although additional experimentation is required to confirm this.

Overall, this study reveals that interactions with the common co-colonizing and co-infecting fungus *C. albicans* lead to activation of two major virulence regulatory systems in *S. aureus,* Agr and SaeRS. SaeRS is required for cytotoxicity toward human monocytes, demonstrating species-specific effects of *S. aureus-C. albicans* interactions. Because *C. albicans* activates SaeRS in *S. aureus*Δ*agr* mutants, clinical isolates with Agr dysfunction may also be susceptible to virulence regulation by *C. albicans* in the context of human infections*.* This study underscores the importance of delineating how *S. aureus* interactions with *C. albicans* impact virulence toward human and murine cells for a more complete and translationally impactful view of how polymicrobial interactions shape *S. aureus* disease.

## Materials and methods

### Ethics statement

All experiments involving animals were reviewed and approved by either the Institutional Animal Care and Use Committee of Vanderbilt University Medical Center (VUMC) or St. Jude Children’s Research Hospital and performed according to NIH guidelines, the Animal Welfare Act, and US Federal Law. Human primary peripheral blood mononuclear cells were commercially sourced from Zen-Bio (a BioIVT company), a commercial vendor that de-identifies their products so donor personal information is not publicly available. Primary human neutrophils were isolated from peripheral whole blood obtained with written consent from healthy donors by researchers within the Vanderbilt Vaccine Research Program, according to the IRB protocol 070258.

### Microbial strains and culture conditions

Strain information for *S. aureus* can be found in S1 Table and for *C. albicans* in S2 Table. Experiments were performed with *S. aureus* USA300 lineage strain LAC* (AH1263), an erythromycin- and tetracycline-sensitive derivative of LAC, as the wild type (WT) strain [49]. All *S. aureus* strains used in this study are in this strain background, except for the clinical isolate strains (VUSA), which were obtained in a de-identified fashion from a separate IRB-approved study. Mutants generated in this study were created using phi-85-mediated phage transduction. Specifically, *agrA::bursa* (Δ*agrA*) from the NARSA strain collection was transduced into Δ*lukAB*, *lukSF-PV::spc* (Δ*pvl*)*,* and *hlgACB::tet* (Δ*hlg*) strains. Additionally, *agrBDCA::tet* (Δ*agr*) was transduced into the P_*saeP*_-*gfp* (*attC*::P_sarA_-*sod*RBS*-mCherry* SAUSA300_RS05730::P_saeP_-*gfp*) and P_*blank*_-*gfp* (*attC*::P_sarA_-*sod*RBS*-mCherry* SAUSA300_RS05730::*gfp*) strains. *S. aureus* Δ*pvl* and each of the *S. aureus* clinical isolates were also transduced with *agrBDCA::tet* (Δ*agr*) to generate Δ*agr*Δ*pvl* and VUSA Δ*agr* strains, respectively. Complementation of Δ*agr*Δ*pvl* was done using pJC1111-p*pvl-pvl,* which expresses *lukSF-PV* from its native promoter [85]. This complementation construct was transduced into *S. aureus* Δ*agr*Δ*pvl,* generating Δ*agr*Δ*pvl + pvl* (*agrBDCA::tet lukSF-PV::spc* SaPI1 *attC::*p*pvl-pvl*). *C. albicans* strain SC5314 was used in all experiments as the wild type reference strain [50]. *C. albicans* clinical isolates were obtained from the *micro*VU biorepository at VUMC in a de-identified fashion. For routine culturing, *S. aureus* strains were grown on tryptic soy agar (TSA) plates before inoculation into 5 mL tryptic soy broth (TSB) in 15 mL conical tubes, which were grown overnight with shaking at 180 rpm and incubation at 37°C. Erythromycin (10 µg/mL), tetracycline (2 µg/mL), spectinomycin (100 µg/mL), or cadmium chloride (0.1 mM) were added to cultures for strains that possess the corresponding resistance cassettes. For routine culturing, *C. albicans* strains were grown first on yeast peptone dextrose (YPD) agar plates and then inoculated in 5 mL YPD broth in 50 mL Erlenmeyer flasks with shaking at 200 rpm and incubation at 30°C overnight.

### Generating *S. aureus* SaeR site binding mutant and revertant strains

Construction of a strain harboring a SaeR-binding site mutation upstream of *pvl* and the associated revertant strain were created as described previously [86]. Briefly, the SaeR binding motif sequence upstream of *pvl*, GTTAA(N_6_)TTAA, was mutated to GGCGG(N_6_)GGAG by overlapping PCR using primers pvl1F (5’-ATGGAATTCGAATCCGCCAGTGCCAGC-3’), pvl2R (5’-CATTTCCTTTCTTTATAAATTTTATTACATTTTTATACTCCACCTTTCCGCCTTTTAATAAAATTAA-3’), pvl3F (5’-TAATTTTATTAAAAGGCGGAAAGGTGGAGTATAAAAATGTAATAAAATTTATAAAGAA-3’), and pvl4R (5’-ATGGGTACCGAAGGATTGAAACCACTGTGTAC-3’) and *S. aureus* wild type genomic DNA as the template. The mutated fragment was cloned into the pKOR1 vector at the *EcoRI* and *KpnI* restriction sites, and allelic replacement on the chromosome of *S. aureus* wild type was performed as described previously [87], generating the binding site mutant (designated *pvl*^*sbm*^). To generate the revertant strain, a wild type fragment was amplified from *S. aureus* wild type via PCR using primers pvl1F and pvl4R. The wild type fragment was cloned into pKOR1 at the *EcoRI* and *KpnI* restriction sites. Allelic replacement was performed in the *pvl*^*sbm*^ strain, generating the revertant strain (designated *pvl*^*rev*^). Successful mutation of the SaeR binding site and reversion back to wild type was confirmed by PCR and sequencing. Following confirmation, *S. aureus pvl*^*sbm*^ and *pvl*^*rev*^ were transduced with *agrBDCA::tet* (Δ*agr*) using phi-85 phage, generating *S. aureus* Δ*agr/pvl*^*sbm*^ and Δ*agr/pvl*^*rev*^.

### Mono- and co-culture supernatant preparation

Overnight cultures of *S. aureus* and *C. albicans* were pelleted and washed once in equal volume of 1X phosphate buffered saline (PBS). Cells were pelleted and resuspended in equal volume PBS for *S. aureus* or half-volume PBS for *C. albicans*. To set up co-cultures, *S. aureus* and *C. albicans* cells were diluted 1:100 in 5 mL RPMI-g, which is RPMI1640 (Gibco) supplemented with 1% casamino acids and 2% D-glucose. Mono-cultures were generated by 1:100 dilution of either *S. aureus* or *C. albicans* in 5 mL RPMI-g. Cultures were incubated in 50 mL conical tubes for 15 hours at 37°C with shaking at 180 rpm. A portion of culture was removed before and after 15 hours and plated on selective media to determine microbial CFU. For growth curves, a portion of culture was removed and plated every 3 hours over 15 hours of growth. After 15 hours growth, cultures were centrifuged at 4000 rpm for 10 minutes to pellet microorganisms. Cultures were kept on ice while each supernatant was filter-sterilized through a 0.22 µm filter into a new tube. Filter-sterilization was repeated, and a portion of the culture supernatant was plated to confirm there was no microbial growth. Supernatants were aliquoted and used either immediately or stored at -80°C until use in cytotoxicity assays.

### Isolation and culture of primary cells

Murine bone marrow-derived monocytes (BMDM) were differentiated from whole bone marrow isolated from wild type C57BL/6J female mice aged 8–12 weeks, as described previously [88]. Cells were resuspended in complete MEM-α medium, which is MEM-α (Gibco) supplemented with 10% fetal bovine serum and 1X penicillin/streptomycin cocktail (Corning). Complete MEM-α was supplemented with 5% supernatant derived from the CMG 14–12 cell line as an M-CSF source. BMDM were seeded in 96-well tissue culture plates at a concentration of 5.0x10^4^ cells per well two days prior to use in cytotoxicity assays. Primary CD14^+^ human monocytes were isolated from fresh peripheral blood mononuclear cells (PBMC) from healthy human donors (ZenBio). Cells were either used upon arrival for negative selection or aliquoted and stored frozen in CryoStor cell preservation media (Sigma) until selection. Negative selection for CD14^+^ monocytes was performed using the Classical Monocyte Isolation Kit, Human (Miltenyi) and following manufacturer instructions. Monocytes were resuspended in complete MEM-α media supplemented with 50 ng/mL of recombinant human M-CSF (R&D Systems). For cytotoxicity assays, 96-well tissue culture plates were seeded with 5.0x10^4^ cells per well. For SYTOX imaging assays, µ-Slide 8 Wells (Ibidi) were seeded with 1.5x10^5^ cells per chamber. A media change was performed 3 days after seeding, and cells were used in cytotoxicity or SYTOX assays 6 days after seeding. Primary neutrophils were isolated from peripheral blood of healthy human donors as previously described [89]. Neutrophils were resuspended in RPMI1640 (Gibco) and 96-well tissue culture plates were seeded at a concentration of 1.0x10^5^ cells per well. Isolated neutrophils were used immediately in the cytotoxicity assay. All primary cells were maintained at 37°C with 5% CO_2_.

### Culture of cell lines

A549 cells were obtained from the American Type Culture Collection (ATCC) and primary human lung microvascular endothelial cells (HLMVEC) were obtained from Promocell. All cells were propagated according to manufacturer recommendations. A549 cells were cultured in Dulbecco’s MEM (DMEM) supplemented with 10% FBS and 1X penicillin/streptomycin, and HLMVEC were cultured in Endothelial Cell Growth Medium MV2 (Promocell). Media was exchanged every 2–3 days for both cell types. Two days prior to cytotoxicity assays, A549 cells were seeded in 96 well tissue culture plates at a density of 1.5x10^4^ cells per well. Prior to use in cytotoxicity assays, HLMVEC were seeded in 96 well plates coated with Attachment Factor (Cell Applications, Inc), and cells were incubated for another 2–3 days to allow for the formation of tight junctions. All cells were grown or maintained at 37°C and 5% CO_2_.

### Supernatant cytotoxicity assay

Culture medium was removed from each well and replaced with fresh media (without added FBS or penicillin/streptomycin) for each cell type. MEM-α for BMDM and CD14^+^ was supplemented with an M-CSF source. Where indicated, CD14^+^ monocytes were treated with 10 µM NDT9513727 (R&D Systems) or an equal volume of DMSO for 15 minutes prior to the addition of supernatants. Two hours after the addition of fresh media, culture supernatant or RPMI-g medium was added to triplicate wells at either a 25% (A549) or 20% (all other cell types) vol/vol concentration. Prior to each time point, cells in three untreated wells were lysed with 0.4% Triton X-100. Plates were returned to incubate at 37°C with 5% CO_2_ for 30 minutes, then samples from each well were diluted 1:25 in LDH Storage Buffer (200 mM Tris/HCl pH 7.3, 10% glycerol, 1% BSA). Samples were either used immediately or stored at -80°C until use. LDH release was quantified using the LDH-Glo Cytotoxicity Assay (Promega) according to manufacturer instructions. Percent LDH release was calculated for each sample by standardizing against LDH values from the Triton X-100 treated wells.

### SYTOX imaging assay

Culture medium was removed from each chamber of CD14^+^ cells that were isolated and seeded in µ-Slide 8 Well chambers as described above. SYTOX Green Nucleic Acid Stain (ThermoFisher Scientific) was diluted to a concentration of 30 nM in fresh MEM-α media (no FBS or penicillin/streptomycin) with M-CSF. Media from each chamber was replaced with SYTOX media, and culture supernatant or RPMI-g was added to replicate chambers at a 20% vol/vol concentration. Using a BioTek Cytation 5 (Agilent), the µ-Slide was kept at 37°C and 5% CO_2_, and three randomly selected fields of view in each chamber were imaged every hour over the course of 15 hours. Both phase contrast and GFP fluorescence (excitation/emission 469/525) were imaged. The Cellular Analysis Cell Counting function on Gen5 software (Agilent, version 3.16) was used to automatically enumerate the number of GFP+ nuclei in each image. Counts for GFP+ nuclei in the three fields of view of each well were added together, and the sum of GFP+ nuclei were averaged for replicate wells.

### Fluorescent reporter assay

*S. aureus* and *C. albicans* were prepared as described for the supernatant preparation. Using RPMI-g that was generated with RPMI1640 without phenol red (Gibco), *S. aureus, C. albicans,* or both organisms were diluted 1:100 and each culture was added to triplicate wells of a black-walled, clear bottom 96 well plate. Using a BioTek Synergy HT plate reader, each plate was incubated for 20 hours with orbital shaking and incubation at 37°C. Every hour, fluorescence was measured using excitation/emission of 479/520 nm to measure GFP and 579/616 nm to measure mCherry. GFP fluorescence for each *saeP* reporter strain was either blanked against the isogenic *gfp* promoterless control strain or standardized against the constitutive mCherry values in each replicate culture.

### RNA isolation and qRT-PCR

*S. aureus* WT and Δ*agr* were grown with or without *C. albicans* in triplicate cultures in RPMI-g medium, as described for supernatant preparation. At 6 and 12 hours of culture, RNA was isolated from each culture as previously described, using Yellow RNA Lysis Kit tubes (Next Advance) and a Bullet Blender (Next Advance) for cell lysis [90]. RNA was isolated using the RNeasy Mini Kit (QIAGEN), according to the manufacturer’s protocol. Isolated RNA was treated with the TURBO DNA Free Kit (ThermoFisher) and then 1 µg of RNA was converted to cDNA using qScript cDNA SuperMix (QuantaBio), following the manufacturers’ protocols. Quantitative RT-PCR (qRT-PCR) was performed using iQ SYBR Green Supermix (Bio-Rad), cDNA, and each primer pair. Primers to quantify *saeP* transcript levels are found in [91]. Primers to quantify *lukSF-PV* transcript levels are 5’-TCTCAAAACAAATGACCCCAAT-3’ and 5’-GGACCACTATTAAAATTACCACC-3’, and primers to quantify *gyrB* transcript levels are 5’-GGATCGACTTCAGAGAGAGG-3’ and 5’-CGTCCGTTATCCGTTACTTTAATC-3’. RNA from each sample without added qScript was used as a negative control for qRT-PCR. Reactions were performed using a CFX96 qPCR cycler (Bio-Rad) and following the manufacturer’s guidelines with an annealing temperature of 52°C. Fold change was calculated using the ΔΔC_t_ method [92], whereby the C_t_ value for each gene was averaged from three technical replicates and standardized against *gyrB* C_t_ values from the same sample.

### DNA isolation, whole genome sequencing, and variant calling of *S. aureus* isolates

Overnight bacterial cultures of *S. aureus* were pelleted and resuspended in lysis buffer [93]. Cells were transferred to Yellow RNA Lysis Kit tubes (Next Advance) and were lysed for 1 min at maximum power in a Bullet Blender (Next Advance). Lysed samples were treated with 100µg/mL RNase A and genomic DNA was extracted using the DNeasy Blood & Tissue Kit (QIAGEN), following manufacturer’s instructions. Isolated DNA quality and concentration were confirmed by Nanodrop and Qubit (ThermoFisher). Whole genome sequencing was performed by SeqCoast Genomics (Portsmouth, New Hampshire) using Illumina short-read sequencing to produce 150 bp paired-end reads. Raw reads were trimmed using BBDuk [94] and mapped to the reference genome *S. aureus* USA300_FPR_3757 (NCBI accession number NC_007793.1) using Geneious Prime v.2025.0.3. Variant calling was performed in Geneious for polymorphisms with a minimum 90% variant frequency and a minimum coverage of 30 reads.

### Peritoneal exudate cell cytotoxicity

Female hC5aR1^KI^ or wild type (WT) C57BL/6J mice aged 12–16 weeks were administered 2 mL sterile 3% Brewer Thioglycolate medium via intraperitoneal injection. At 24 hours post injection, peritoneal exudate cells (PEC) were collected via a lavage of the peritoneal cavity with 10 mL cold PBS. PEC were centrifuged at 500 x *g* for 10 min, then red blood cells were lysed with ACK lysis buffer. Cells were washed and resuspended in RPMI1640 media supplemented with 10% FBS. PEC were seeded at a density of 2.5x10^5^ cells per well in a 96 well plate and treated with 1 µg/mL or 10 µg/mL of recombinant purified PVL [95]. PBS was used as a mock treatment. Following 1 hour of incubation at 37C with 5% CO2, cells were collected by centrifugation and washed in FACS buffer twice. PEC were incubated for 15 min at room temperature with 5% TruStain FcX mouse anti CD16/32 (BioLegend) containing fixable viability dye eFluor660 (1:800, eBioscience). Following two washes with FACS buffer, cells were stained with PerCP anti-mouse CD45 (1:100, clone 30-F11, BioLegend), PE anti-mouse/human CD11b (1:50, clone M1/70, BioLegend) for 20 min at room temperature. Cells were washed with FACS buffer twice then acquired on a Cytek Northern Lights flow cytometer and the data analyzed with FlowJo version 10.1. Gating was performed to select singlets and then CD11b^+^CD45^+^ cells. Viability was determined based on the proportion of eFluor660^+^ (dead) vs. eFluor660^-^ (live) cells.

### Murine infection

Female hC5aRI^KI^ mice aged 12–16 weeks were inoculated via intraperitoneal injection of 100 µL of *C. albicans, S. aureus,* or co-culture of both organisms grown as described for supernatant preparation. *S. aureus* strains were inoculated at 1.3-3.0x10^8^ CFU and *C. albicans* at 1.0-1.9x10^6^ CFU. Mice were monitored daily over the course of four days post inoculation for morbidity and mortality. Disease score was based on weight loss and the observation of reduced mobility, hunched posture, and ruffled fur. Mice receiving a score of 4 or above prior to the end of the experiment were humanely euthanized. All mice were euthanized at the end of the experiment.

### Statistical analysis

Statistical analyses were performed using Prism (GraphPad, version 10.5.0). Data were checked for normality prior to statistical analysis. To assess the effects of co-culture and strain on cytotoxicity toward host cells, a one-way ANOVA was used with a *post hoc* Tukey’s multiple comparisons test. To assess the effects of co-culture and reporter construct on fluorescence levels, a one-way ANOVA was used with a *post hoc* Tukey’s multiple comparisons test. To assess the effects of co-culture on transcript levels, a one-way ANOVA was used with a *post hoc* Dunnett’s test of multiple comparisons, to compare relative to wild type mono-culture transcript levels. To assess the effects of *C. albicans* co-infection on *S. aureus* virulence, a Log-Rank (Mantel-Cox) test was used.

## Supporting information

S1 Fig *S. aureus* and *C. albicans* growth is similar in mono-culture and co-culture. *S. aureus* wild type and *C. albicans* were cultured together or separately in RPMI-g (RPMI1640 supplemented with 1% casamino acids and 2% D-glucose) for 15 hours. Every 3 hours, an aliquot of culture was removed and plated on selective media to enumerate CFU/mL for *S. aureus* and *C. albicans.* Red circles represent *S. aureus* CFU/mL and blue squares represent *C. albicans* CFU/mL. Open symbols represent microbial burdens from mono-cultures and filled symbols represent microbial burdens from co-cultures. N = 3 replicates per culture and error bars represent SD. Some error bars are hidden by the symbols.
(PDF)

S2 Fig Single mutants for genes encoding α-toxin and γ-hemolysin retain cytotoxicity towards murine monocytes. Murine bone marrow-derived monocytes were exposed to culture supernatants from *S. aureus* strains grown with or without *C. albicans.* % LDH release was quantified at 24 hours after supernatant exposure. *S. aureus* WT = wild type; Δ*agr* = *agrBDCA::tet*; Δ*hla* = *hla*::*spc*; Δ*hlg* = *hlgACB::tet*. The first three conditions (media only, WT mono-culture, and WT co-culture) are the same data presented in Fig 1C. ****p < 0.001 and ns (not significant) by one-way ANOVA with Tukey’s multiple comparisons test; n = 3 wells per supernatant condition, bars = mean ± SD.
(PDF)

S3 Fig Human monocyte cell death occurs rapidly after exposure to supernatant from *S. aureus* Δ*agr* co-cultured with *C. albicans.* Human CD14^+^ monocytes in media containing SYTOX Green nucleic acid stain were exposed to culture supernatants from *S. aureus* Δ*agr* grown with or without *C. albicans.* (A) Using the Gen5 Cellular Analysis Cell Counting function, SYTOX+ nuclei were enumerated in from three randomly chosen fields of view in each well every hour over 15 hours of imaging. N = 3 replicate wells per condition, error bars represent SD. Symbols for the RPMI and Δ*agr* group are overlapping. (B-I) Representative images of monocytes at 0hr (B,F), 2hr (C,G), 4hr (D,H), and 6hr (E,I) after exposure to supernatant from *S. aureus* Δ*agr* mono-culture (B-E) or *S. aureus* Δ*agr* co-cultured with *C. albicans* (F-I).
(PDF)

S4 Fig *C. albicans* co-culture does not enhance constitutive mCherry fluorescence in *S. aureus.* *S. aureus* wild type and Δ*agr P*_*saeP*_-*gfp* (*attC*::P_sarA_-*sod*RBS*-mCherry* SAUSA300_RS05730::P_saeP_-*gfp*) or *P*_*blank*_*-gfp* (*attC*::P_sarA_-*sod*RBS*-mCherry* SAUSA300_RS05730::*gfp*) strains were cultured with and without *C. albicans* (*C.a.*)*,* and mCherry fluorescence values were measured every hour for 20 hours. N = 3 wells or replicates per condition, and error bars represent SD. Some error bars are hidden by the symbols.
(PDF)

S5 Fig *C. albicans* induces *S. aureus* Δ*agr* cytotoxicity by 12 hours of co-culture growth. Human CD14^+^ monocytes were exposed to supernatant from *S. aureus* strains cultured with and without *C. albicans* for either 6 hours or 12 hours in RPMI-g. % LDH release was quantified at 24 hours after supernatant exposure. *S. aureus* Δ*agr = agrBDCA::tet.* ****p < 0.0001 and ns (not significant) by one-way ANOVA with Tukey’s multiple comparisons test; n = 3 wells or replicates per condition, bars = mean ± SD.
(PDF)

S6 Fig *C. albicans* co-culture enhances cytotoxicity of *S. aureus* single toxin deletion mutants towards primary human monocytes. Human CD14^+^ monocytes were exposed to supernatant from *S. aureus* strains cultured with and without *C. albicans*. % LDH release was quantified at 4 hours (A) or 24 hours (B) after supernatant exposure. *S. aureus* WT = wild type; Δ*pvl = lukSF-PV::spc*; Δ*AB* = Δ*lukAB*; Δ*hlg = hlgACB::tet.* *p < 0.05 and ns (not significant) by one-way ANOVA with Tukey’s multiple comparisons test; n = 3 wells or replicates per condition, bars = mean ± SD.
(PDF)

S7 Fig *S. aureus* LAC* and clinical isolates grown in mono-culture and co-culture trigger maximal cytotoxicity toward human cells by 24 hours. Human CD14^+^ monocytes were exposed to supernatant from *S. aureus* LAC* and sequence type 8 clinical isolate strains cultured with and without *C. albicans*. % LDH release was quantified 24 hours after supernatant exposure. N = 3 wells or replicates per condition, bars = mean ± SD.
(PDF)

S8 Fig *C. albicans* SC5314 and clinical isolates maintain media at a neutral pH and do not alter bacterial growth during co-culture with *S. aureus.* *S. aureus* strains were cultured with and without *C. albicans* for 15 hours in RPMI-g. The media pH and bacterial CFU/mL were measured for *S. aureus* mono-culture and each co-culture for wild type (A and C) and Δ*agr* (B and D). For C and D, n = 3 replicates per culture, and some error bars are hidden by the symbols.
(PDF)

S1 Table *Staphylococcus aureus* strains used in this work. (PDF)

S2 Table *Candida albicans* strains used in this work. (PDF)
