# Supplementary material for: Candida albicans activates Staphylococcus aureus virulence regulatory systems to drive toxin-mediated human cell death
Source: PLoS Pathog. 2026 Aug 5;22(8):e1014490. doi: 10.1371/journal.ppat.1014490 (PMC13475977; doi:10.1371/journal.ppat.1014490)
Supplement: Supplementary file 1 [file ppat.1014490.s001.pdf]

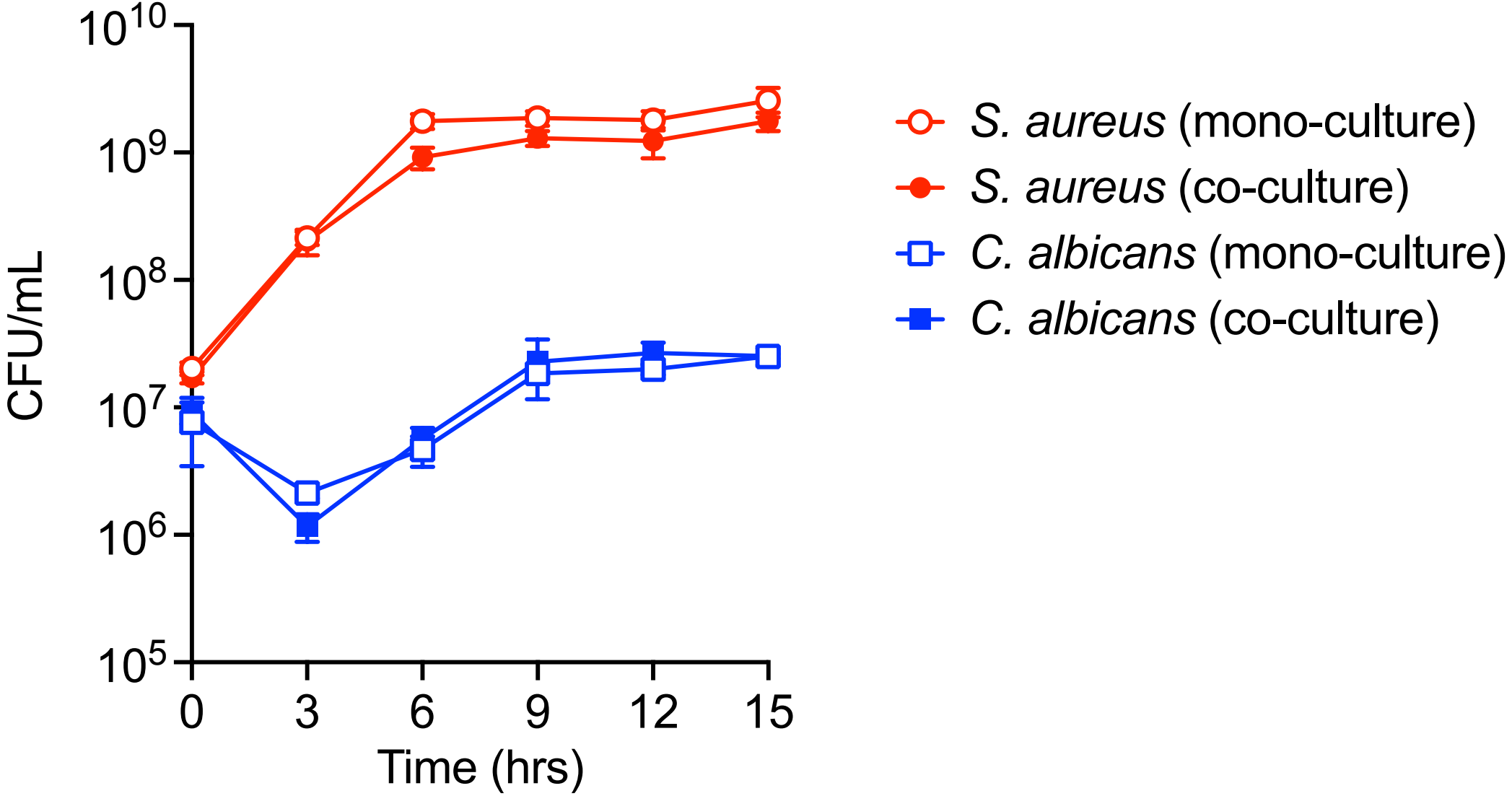

CFU/mL
10^{10}
10^{9}
10^{8}
10^{7}
10^{6}
10^{5}
0
3
6
9
12
15
Time (hrs)
S. aureus (mono-culture)
S. aureus (co-culture)
C. albicans (mono-culture)
C. albicans (co-culture)
