# Supplementary material for: Candida albicans activates Staphylococcus aureus virulence regulatory systems to drive toxin-mediated human cell death
Source: PLoS Pathog. 2026 Aug 5;22(8):e1014490. doi: 10.1371/journal.ppat.1014490 (PMC13475977; doi:10.1371/journal.ppat.1014490)
Supplement: Supplementary file 3 [file ppat.1014490.s003.pdf]

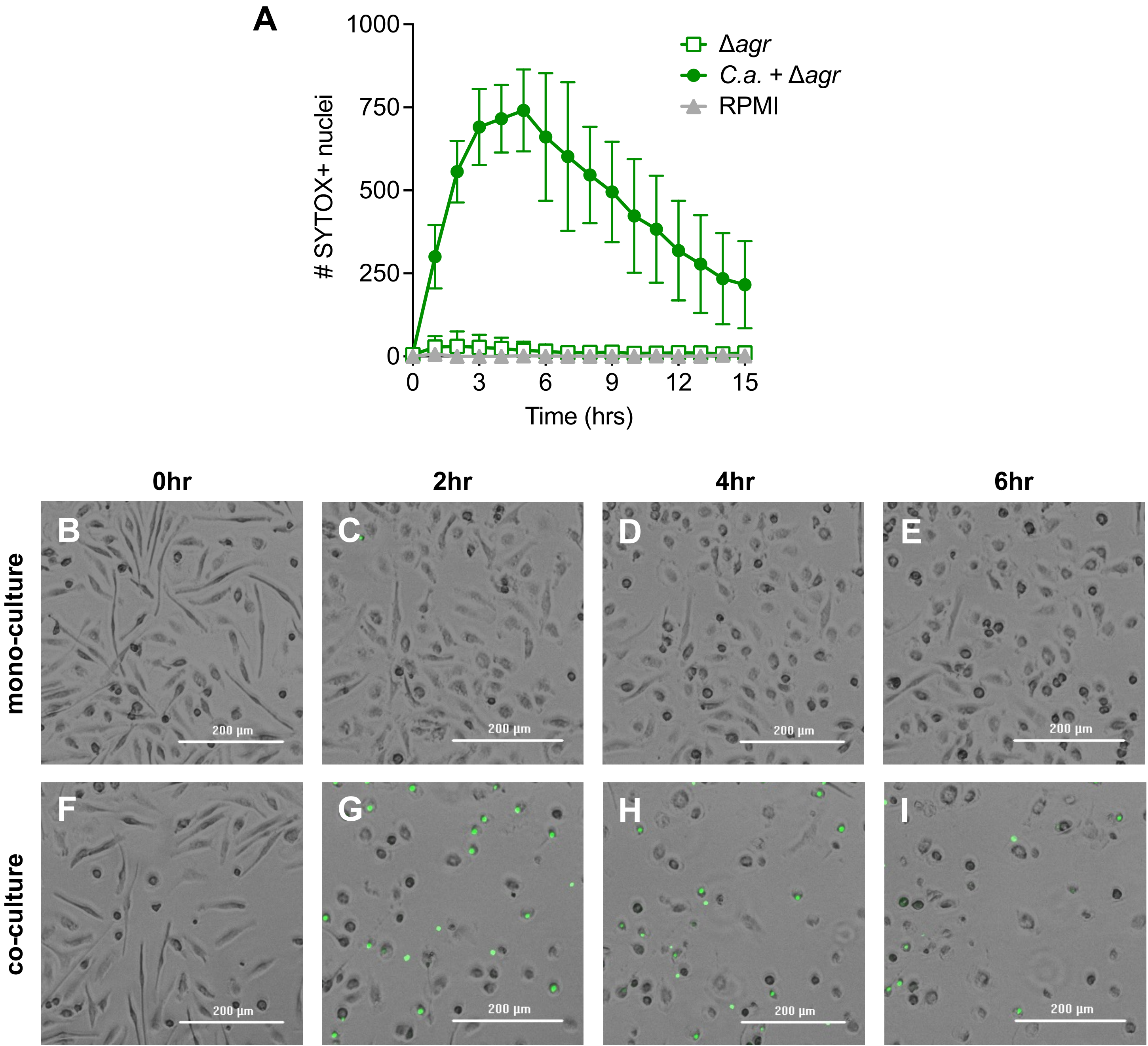

A
1000
750
500
250
0
SYTOX+ nuclei
0
3
6
9
12
15
Time (hrs)
Δagr
C.a. + Δagr
RPMI
0hr
2hr
4hr
6hr
mono-culture
co-culture
B
C
D
E
F
G
H
I
200 µm
