# Supplementary material for: Candida albicans activates Staphylococcus aureus virulence regulatory systems to drive toxin-mediated human cell death
Source: PLoS Pathog. 2026 Aug 5;22(8):e1014490. doi: 10.1371/journal.ppat.1014490 (PMC13475977; doi:10.1371/journal.ppat.1014490)
Supplement: Supplementary file 4 [file ppat.1014490.s004.pdf]

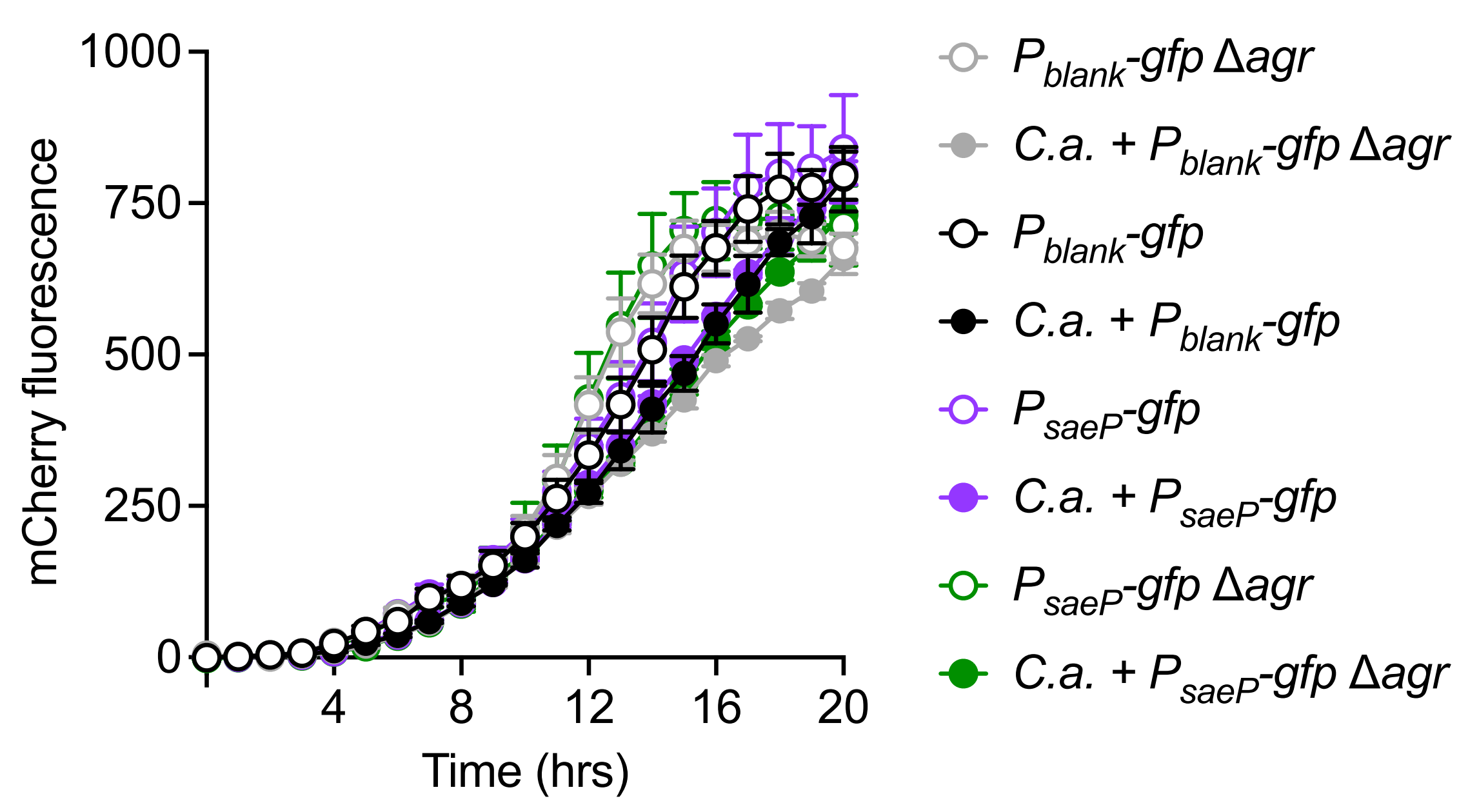
1000
750
500
250
0
4
8
12
16
20
Time (hrs)
mCherry fluorescence
P_blank-gfp Δagr
C.a. + P_blank-gfp Δagr
P_blank-gfp
C.a. + P_blank-gfp
P_saeP-gfp
C.a. + P_saeP-gfp
P_saeP-gfp Δagr
C.a. + P_saeP-gfp Δagr
