# Supplementary material for: Candida albicans activates Staphylococcus aureus virulence regulatory systems to drive toxin-mediated human cell death
Source: PLoS Pathog. 2026 Aug 5;22(8):e1014490. doi: 10.1371/journal.ppat.1014490 (PMC13475977; doi:10.1371/journal.ppat.1014490)
Supplement: Supplementary file 5 [file ppat.1014490.s005.pdf]

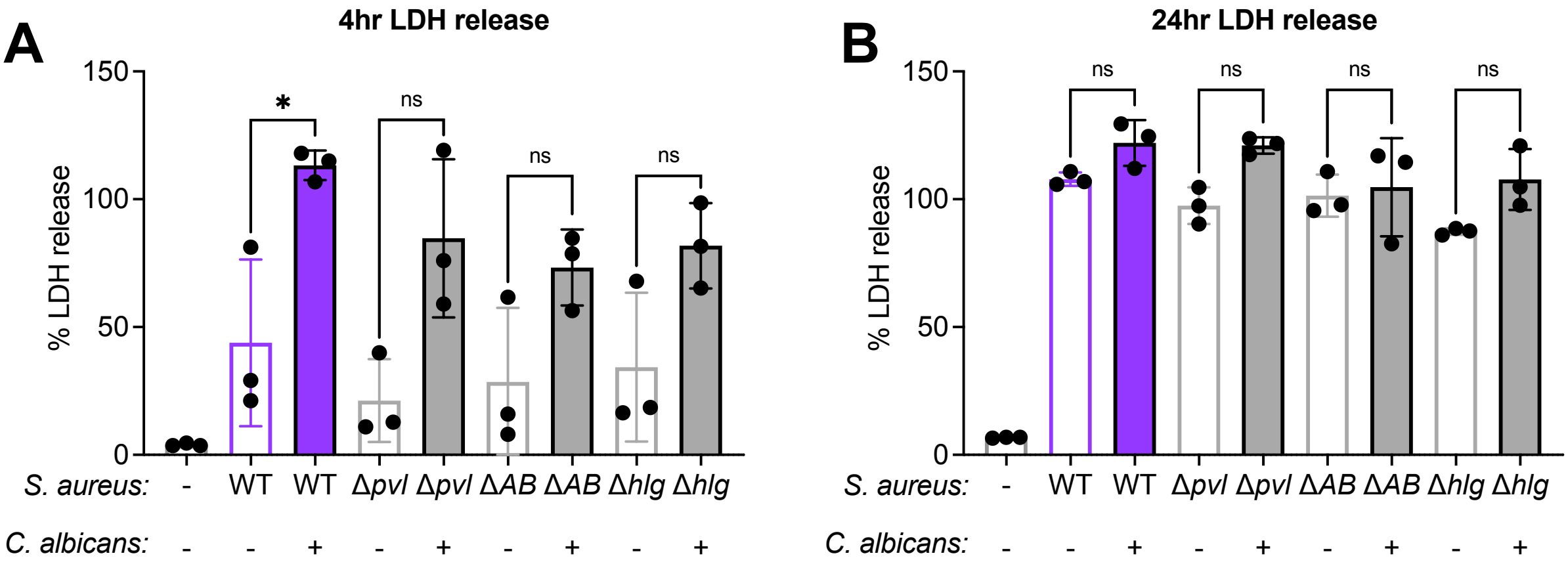
A
4hr LDH release
B
24hr LDH release
% LDH release
150
100
50
0
*
ns
S. aureus: - WT WT Δpvl Δpvl ΔAB ΔAB Δhlg Δhlg
C. albicans: - - + - + - + - +
