# Supplementary material for: Candida albicans activates Staphylococcus aureus virulence regulatory systems to drive toxin-mediated human cell death
Source: PLoS Pathog. 2026 Aug 5;22(8):e1014490. doi: 10.1371/journal.ppat.1014490 (PMC13475977; doi:10.1371/journal.ppat.1014490)
Supplement: Supplementary file 6 [file ppat.1014490.s006.pdf]

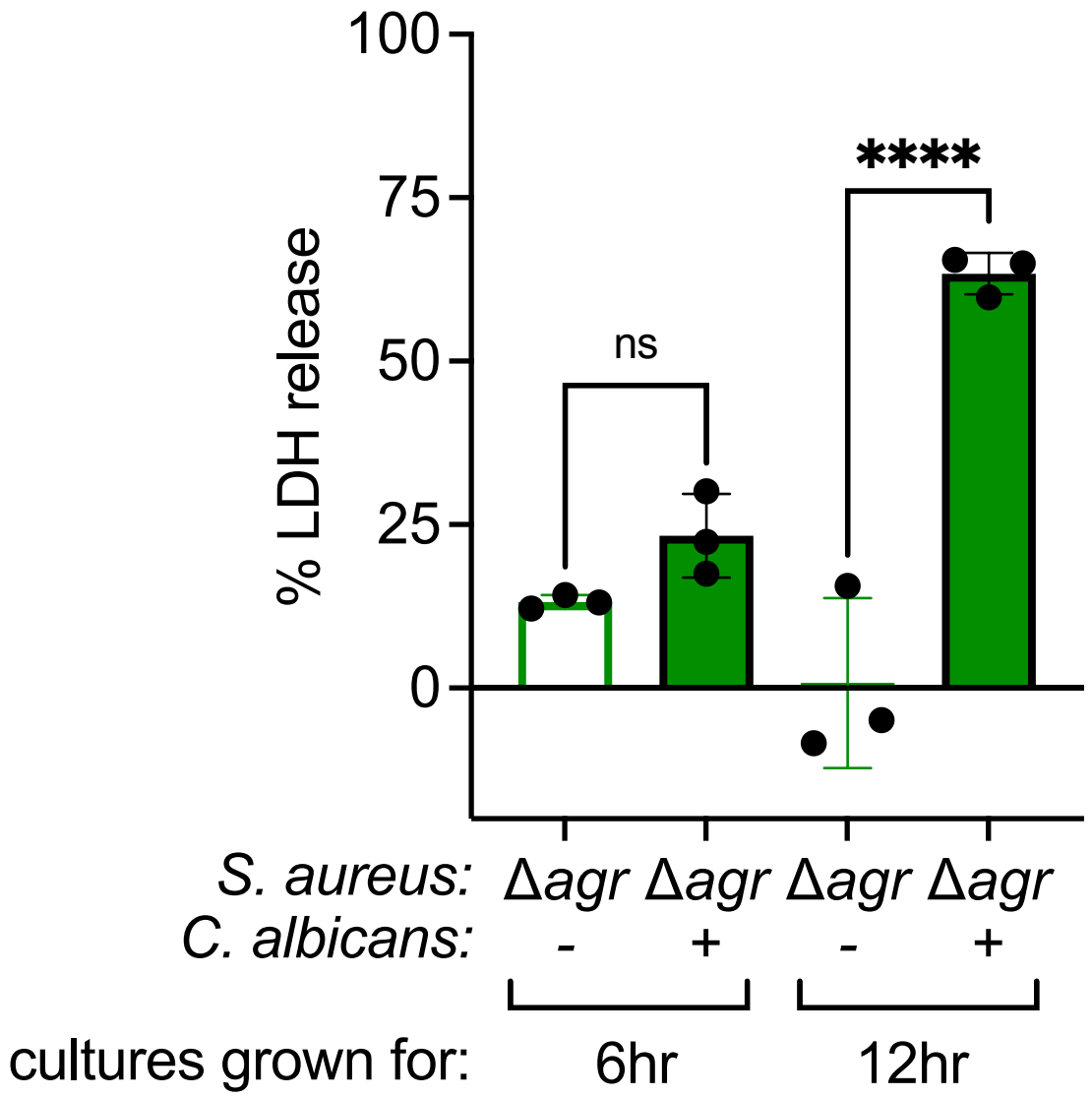

100
75
50
25
0
% LDH release
ns
****
S. aureus:
Δagr
Δagr
Δagr
Δagr
C. albicans:
-
+
-
+
cultures grown for:
6hr
12hr
