# Supplementary material for: Candida albicans activates Staphylococcus aureus virulence regulatory systems to drive toxin-mediated human cell death
Source: PLoS Pathog. 2026 Aug 5;22(8):e1014490. doi: 10.1371/journal.ppat.1014490 (PMC13475977; doi:10.1371/journal.ppat.1014490)
Supplement: Supplementary file 7 [file ppat.1014490.s007.pdf]

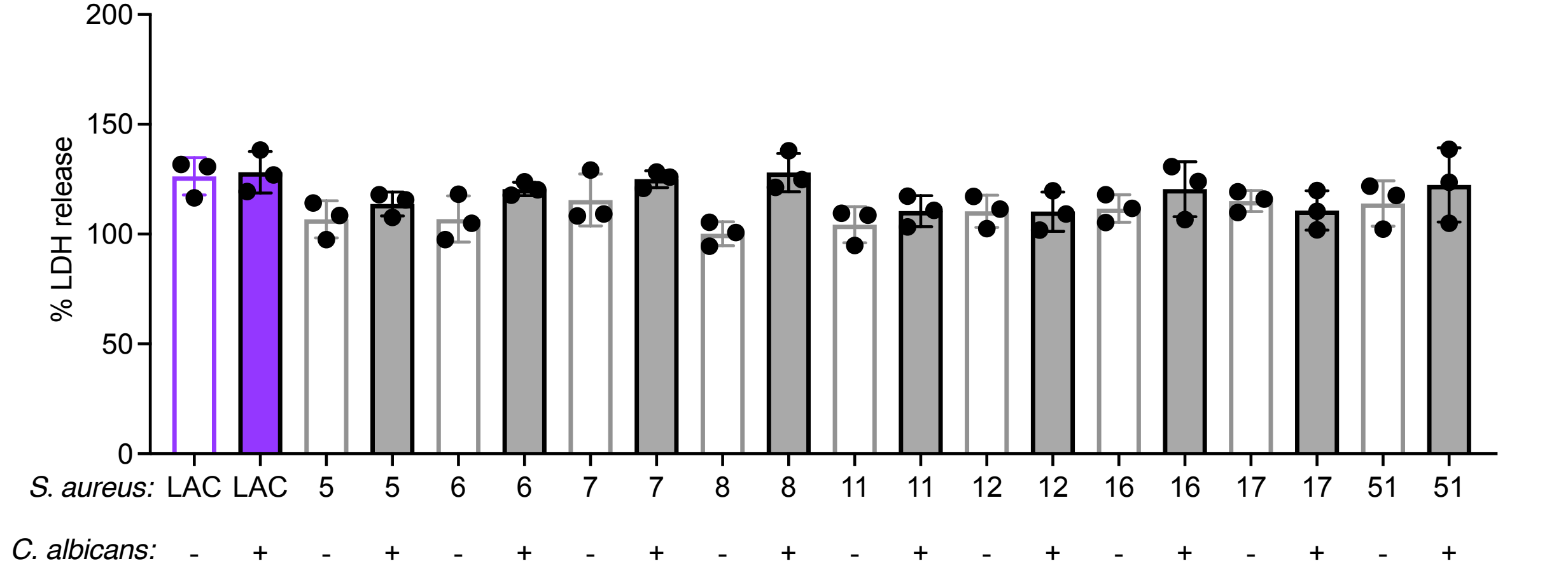

% LDH release
200
150
100
50
0
S. aureus: LAC LAC 5 5 6 6 7 7 8 8 11 11 12 12 16 16 17 17 51 51
C. albicans: - + - + - + - + - + - + - + - + - + - +
