# Supplementary material for: Candida albicans activates Staphylococcus aureus virulence regulatory systems to drive toxin-mediated human cell death
Source: PLoS Pathog. 2026 Aug 5;22(8):e1014490. doi: 10.1371/journal.ppat.1014490 (PMC13475977; doi:10.1371/journal.ppat.1014490)
Supplement: Supplementary file 8 [file ppat.1014490.s008.pdf]

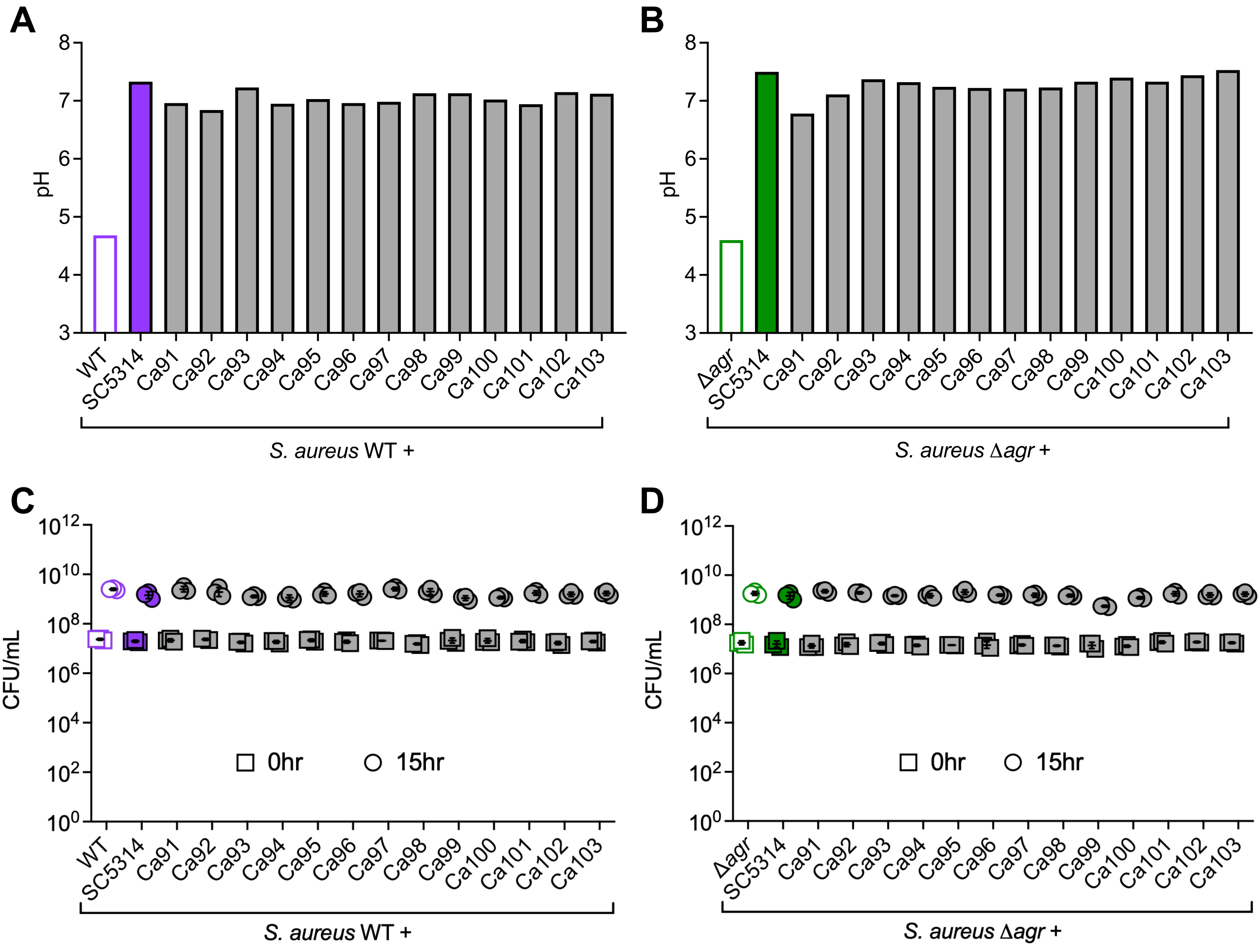

A
pH
8
7
6
5
4
3
WT
SC5314
Ca91
Ca92
Ca93
Ca94
Ca95
Ca96
Ca97
Ca98
Ca99
Ca100
Ca101
Ca102
Ca103
S. aureus WT +
B
pH
Δagr
SC5314
S. aureus Δagr +
C
CFU/mL
10^{12}
10^{10}
10^{8}
10^{6}
10^{4}
10^{2}
10^{0}
0hr
15hr
S. aureus WT +
D
CFU/mL
0hr
15hr
S. aureus Δagr +
