# Supplementary material for: Candida albicans activates Staphylococcus aureus virulence regulatory systems to drive toxin-mediated human cell death
Source: PLoS Pathog. 2026 Aug 5;22(8):e1014490. doi: 10.1371/journal.ppat.1014490 (PMC13475977; doi:10.1371/journal.ppat.1014490)
Supplement: Supplementary file 9 [file ppat.1014490.s009.pdf]

Table S1. *Staphylococcus aureus* strains used in this work.

Strain name	Description/genotype	Source
AH1263 (LAC*)	Wild type reference strain; USA300 MRSA Erm^S	(1)
Δ*agr*	LAC* *agrBDCA::tet*	(2)
Δ*psm*	LAC* *psmα1-4::erm*	(3)
Δ*hla*	LAC* *hla::spc*	(4)
Δ*hlg*	LAC* *hlgACB::tet*	(5)
Δ*psm*Δ*hla*	LAC* *psmα1-4::erm hla::spc*	This study
Δ*psm*Δ*hlg*	LAC* *psmα1-4::erm hlgACB::tet*	This study
Δ*psm*Δ*hlg*Δ*hla*	LAC* *psmα1-4::erm hlgACB::tet hla::spc*	This study
Δ*sae*	LAC* *saeQRS::spc*	(2)
Δ*agr*Δ*sae*	LAC* *agrBDCA::tet saeQRS::spc*	(2)
P_{saeP}-*gfp*	LAC* SaPI1 *attC*::P_{sarA}-*sod*RBS-*mCherry* SAUSA300_RS05730::P_{saeP}-*gfp*	(6)
P_{saeP}-*gfp* Δ*agr*	LAC* SaPI1 *attC*::P_{sarA}-*sod*RBS-*mCherry* SAUSA300_RS05730::P_{saeP}-*gfp agrBDCA::tet*	This study
P_{blank}-*gfp*	LAC* SaPI1 *attC*::P_{sarA}-*sod*RBS-*mCherry* SAUSA300_RS05730::*gfp*	(6)
P_{blank}-*gfp* Δ*agr*	LAC* SapI1 *attC*::P_{sarA}-*sod*RBS-*mCherry* SAUSA300_RS05730::*gfp agrBDCA::tet*	This study
Δ*agrA*	LAC* *agrA::bursa*	NARSA
Δ*pvl*	LAC* *lukSF-PV::spc*	(7)
Δ*lukAB*	LAC* Δ*lukAB*	(8)
Δ*agrA*Δ*pvl*	LAC* *agrA::bursa lukSF-PV::spc*	This study
Δ*agrA*Δ*hlg*	LAC* *agrA::bursa hlgACB::tet*	This study
Δ*agrA*Δ*lukAB*	LAC* *agrA::bursa* Δ*lukAB*	This study
Δ*agr*Δ*pvl*	LAC* *agrBDCA::tet lukSF-PV::spc*	This study
Δ*agr*Δ*pvl* +*pvl*	LAC* *agrBDCA::tet lukSF-PV::spc* SaPI1 *attC::*p*pvl-pvl*	This study
Δ*agr*/pvl^{sbm}	LAC* *agrBDCA::tet;* SaeR binding site mutant	This study
Δ*agr*/pvl^{rev}	LAC* *agrBDCA::tet;* SaeR binding site revertant	This study
VUSA5	ST8 clinical isolate	This study
VUSA6	ST8 clinical isolate	This study
VUSA7	ST8 clinical isolate	This study
VUSA8	ST8 clinical isolate	This study
VUSA11	ST8 clinical isolate	This study
VUSA12	ST8 clinical isolate	This study
VUSA16	ST8 clinical isolate	This study
VUSA17	ST8 clinical isolate	This study
VUSA51	ST8 clinical isolate	This study
VUSA5 Δ*agr*	ST8 clinical isolate *agrBDCA::tet*	This study
VUSA6 Δ*agr*	ST8 clinical isolate *agrBDCA::tet*	This study
VUSA7 Δ*agr*	ST8 clinical isolate *agrBDCA::tet*	This study

VUSA8 Δ*agr*	ST8 clinical isolate *agrBDCA::tet*	This study
VUSA11 Δ*agr*	ST8 clinical isolate *agrBDCA::tet*	This study
VUSA12 Δ*agr*	ST8 clinical isolate *agrBDCA::tet*	This study
VUSA16 Δ*agr*	ST8 clinical isolate *agrBDCA::tet*	This study
VUSA17 Δ*agr*	ST8 clinical isolate *agrBDCA::tet*	This study
VUSA51 Δ*agr*	ST8 clinical isolate *agrBDCA::tet*	This study

References

1. Boles BR, Thoendel M, Roth AJ, Horswill AR. 2010. Identification of Genes Involved in Polysaccharide-Independent *Staphylococcus aureus* Biofilm Formation. *PLoS ONE* 5:e10146.

2. Benson MA, Lilo S, Nygaard T, Voyich JM, Torres VJ. 2012. Rot and SaeRS Cooperate To Activate Expression of the Staphylococcal Superantigen-Like Exoproteins. *J Bacteriol* 194:4355–4365.

3. Cassat JE, Hammer ND, Campbell JP, Benson MA, Perrien DS, Mrak LN, Smeltzer MS, Torres VJ, Skaar EP. 2013. A Secreted Bacterial Protease Tailors the *Staphylococcus aureus* Virulence Repertoire to Modulate Bone Remodeling during Osteomyelitis. *Cell Host Microbe* 13:759–772.

4. Nygaard TK, Pallister KB, DuMont AL, DeWald M, Watkins RL, Pallister EQ, Malone C, Griffith S, Horswill AR, Torres VJ, Voyich JM. 2012. Alpha-Toxin Induces Programmed Cell Death of Human T cells, B cells, and Monocytes during USA300 Infection. *PLoS ONE* 7:e36532.

5. Blake KJ, Baral P, Voisin T, Lubkin A, Pinho-Ribeiro FA, Adams KL, Roberson DP, Ma YC, Otto M, Woolf CJ, Torres VJ, Chiu IM. 2018. *Staphylococcus aureus* produces pain through pore-forming toxins and neuronal TRPV1 that is silenced by QX-314. *Nat Commun* 9:37.

6. Anil A, Braza RED, Liu B, Altouma V, Adedeji C, Welling A, Ellison AL, Check MH, Irnov I, Prescott R, Torres VJ, Davis KM. 2025. *Staphylococcus aureus* exhibits spatiotemporal heterogeneity in Sae activity during kidney abscess development. *mBio* 16:e02043-25.

7. Yoong P, Torres VJ. 2015. Counter inhibition between leukotoxins attenuates *Staphylococcus aureus* virulence. *Nat Commun* 6:8125.

8. DuMont AL, Yoong P, Day CJ, Alonzo F, McDonald WH, Jennings MP, Torres VJ. 2013. *Staphylococcus aureus* LukAB cytotoxin kills human neutrophils by targeting the CD11b subunit of the integrin Mac-1. *Proc Natl Acad Sci USA* 110:10794–10799.
