# Supplementary material for: Candida albicans activates Staphylococcus aureus virulence regulatory systems to drive toxin-mediated human cell death
Source: PLoS Pathog. 2026 Aug 5;22(8):e1014490. doi: 10.1371/journal.ppat.1014490 (PMC13475977; doi:10.1371/journal.ppat.1014490)
Supplement: Supplementary file 10 [file ppat.1014490.s010.pdf]

**Table S2.** *Candida albicans* strains used in this work.

| Strain name | Description/genotype | Source |
|---|---|---|
| SC5314 | Wild type reference strain | (1) |
| pKE4-NRG1 | *ura3Δ/Δ:URA3*-Pr*TEF1-NRG1* | (2) |
| *als3Δ/Δ* | *iro1-ura3Δ::λimm*$^{434}$ */iro1-ura3Δ::λimm*$^{434}$ *als3laΔ/als3saΔ-URA3* | (3) |
| *stp2Δ/Δ* | *stp2Δ:FRT stp2Δ:FRT* | (4) |
| *zcf13Δ/Δ* | *zcf13Δ:FRT zcf13Δ:FRT NEUT5L/NEUT5L* | (5) |
| Ca91 | Clinical isolate from the respiratory tract | This study |
| Ca92 | Clinical isolate from the respiratory tract | This study |
| Ca93 | Clinical isolate from the respiratory tract | This study |
| Ca94 | Clinical isolate from the respiratory tract | This study |
| Ca95 | Clinical isolate from the respiratory tract | This study |
| Ca96 | Clinical isolate from the respiratory tract | This study |
| Ca97 | Clinical isolate from the respiratory tract | This study |
| Ca98 | Clinical isolate from the respiratory tract | This study |
| Ca99 | Clinical isolate from the respiratory tract | This study |
| Ca100 | Clinical isolate from the respiratory tract | This study |
| Ca101 | Clinical isolate from the respiratory tract | This study |
| Ca102 | Clinical isolate from the respiratory tract | This study |
| Ca103 | Clinical isolate from the respiratory tract | This study |

**References**


1. Gillum AM, Tsay EYH, Kirsch DR. 1984. Isolation of the *Candida albicans* gene for orotidine-5′-phosphate decarboxylase by complementation of *S. cerevisiae ura3* and *E. coli pyrF* mutations. *Mol Gen Genet* 198:179–182.

2. Reitler P, Regan J, DeJarnette C, Srivastava A, Carnahan J, Tucker KM, Meibohm B, Peters BM, Palmer GE. 2024. The atypical antipsychotic aripiprazole alters the outcome of disseminated *Candida albicans* infections. *Infect Immun* 92:e00072-24.

3. Zhao X, Oh S-H, Cheng G, Green CB, Nuessen JA, Yeater K, Leng RP, Brown AJP, Hoyer LL. 2004. ALS3 and ALS8 represent a single locus that encodes a *Candida albicans* adhesin; functional comparisons between Als3p and Als1p. *Microbiology* 150:2415–2428.

4. Vylkova S, Lorenz MC. 2014. Modulation of Phagosomal pH by *Candida albicans* Promotes Hyphal Morphogenesis and Requires Stp2p, a Regulator of Amino Acid Transport. *PLoS Pathog* 10:e1003995.

5. Paul S, Todd OA, Eichelberger KR, Tkaczyk C, Sellman BR, Noverr MC, Cassat JE, Fidel PL, Peters BM. 2024. A fungal metabolic regulator underlies infectious synergism during *Candida albicans-Staphylococcus aureus* intra-abdominal co-infection. *Nat Commun* 15:5746.
