# Supplementary material for: Candida albicans activates Staphylococcus aureus virulence regulatory systems to drive toxin-mediated human cell death
Source: PLoS Pathog. 2026 Aug 5;22(8):e1014490. doi: 10.1371/journal.ppat.1014490 (PMC13475977; doi:10.1371/journal.ppat.1014490)
Supplement: Supplementary file 11 [file ppat.1014490.s012.docx]

**Response to Reviewers**

We would like to thank the three reviewers for their critiques and thoughtful suggestions, which we have addressed by revisions to the text and figures. A point-by-point response to the reviewers’ critiques is provided below, in the blue text.

**Major Issues:**

**Reviewer #1:**

The group’s 2024 paper (<https://doi.org/10.1038/s41467-024-50058-w>) shows that ribose consumption by *C. albicans* activates Agr in *S. aureus*. This is an easy experiment to demonstrate that the phenotype observed with SaeRS is independent of the previously described ribose repression.

We thank the reviewer for this suggestion, and we have now added additional data testing the zcf13 mutant that was previously shown to be defective for enhanced Agr activation. We have also included testing several additional *C. albicans* mutants that have been shown to have defects in interactions with *S. aureus.* These new data are presented in panel C of Figure 6 (previously Figure 7) and provide further support that *C. albicans* enhances S. aureus cytotoxicity toward human cells via a mechanism different from those previously described.

It appears that the enhancement of virulence via the Agr system has a larger functional effect (PSMs, Hlg, PVL all regulated by Agr). Therefore, the SaeRS mediated mechanism is only relevant in situations where the Agr system is deficient or not activated. The authors have not made a clear and compelling case regarding the clinical relevance of this scenario.

To address clinical relevance of *C. albicans* enhancing virulence of *S. aureus agr* mutants, we added new data from in vivo infections using a transgenic mouse model that expresses the human isoform of the PVL receptor, C5aR1. We observe enhanced mortality during co-infection relative to mono-infection, and additional data suggests that disease severity requires PVL. These data are in the new Figure 7.

While there are some niches that encourage selection of Agr mutations, such as implant associated biofilm infections, is there any evidence to suggest this regularly occurs in polymicrobial infections, such as chronic wounds?

We include new text in the Discussion section (lines 473-488) that provides additional rationale for the potential of *Candida* to enhance virulence of *S. aureus agr* dysfunctional strains during polymicrobial infection. While it is not clear whether polymicrobial infections themselves induce selection for *S. aureus* *agr* mutants*,* there is significant overlap in host niche for sites of polymicrobial infections and sites where strains with *agr* dysfunction are isolated. Reduced *agr* activity could occur through either *agr* mutation or decreased *agr* activity via reduced quorum sensing or other metabolic cues. We propose that such *S. aureus* isolates are still susceptible to virulence regulation by *C. albicans*.

Unlike in other figures, in Figure 7, there are no controls to demonstrate that the degree of *S. aureus* cytotoxicity enhancement (or lack of) is not due to differences in *S. aureus* growth.

We now include additional panels (B and C) to Figure S8 confirming that *S. aureus* growth is similar among the different co-cultures tested in this figure (previously Figure 7, now Figure 6 for this revised manuscript).

**Minor Issues:**

**Reviewer #1:**

In Figure S3 the caption states that samples taken every 30 minutes but the graph appears to only show data points each hour.

The Figure S3 caption and the associated text in the methods (line 668) has been corrected to be every hour.

Line 194: “These data support that both Agr and SaeRS-regulated toxins are required for human cell death.” I disagree with the phrasing of this sentence. The data in Figure 3a show that *C. albicans* can enhance toxicity through either SaeRS or Agr. Furthermore, PVL is known to be regulated by both SaeRS and Agr, so signaling through either system could enhance PVL cytotoxicity. These data also do not distinguish whether PVL is the only toxin that is sufficient for human cell death or if Agr-regulated α-PSMs also will cause human cell death.

We agree with the reviewer that the original phrasing of this statement was not reflective of the main point of the Figure 3A. This line (now 230-232) has been updated with the suggested text, to more accurately convey the data presented. PVL is regulated through both Agr and SaeRS. Data presented in Figure 4 demonstrate that PVL is the main toxin driving human cell death induced by *C. albicans* in the absence of *agr.* However, PVL is not the only toxin sufficient for the Agr-regulated human cell death, as a single PVL mutant is still cytotoxic towards human cells and this can be enhanced by *Candida*, as demonstrated by the data in Figure S6.

In Figure 7, I would think that the more important statistical test for these two figures would be to compare the clinical strains to the mono-culture to assess whether all clinical strains can significantly increase *S. aureus* cytotoxicity. As it is, the statistical analysis appears to ask whether each strain induces more or less cytotoxicity than the reference strain, which is a slightly different question than what is framed in the paper (Line 291- “These data indicate that the ability to enhance *S. aureus* cytotoxicity is broadly conserved among *C. albicans* isolates…”).

Because we comment upon both comparisons in the text of the manuscript, we updated this figure as suggested (previously Figure 7, now Figure 6, panels A and B) to include statistical comparison of all co-cultures relative to mono-culture and all clinical isolate co-cultures relative to co-culture with SC5314, the laboratory reference strain.

**Reviewer #2:**

This manuscript is well-written and easy to understand. There are only some minor comments:

The authors use “wild-type” in the entire manuscript, which is an adjective and should be replaced by “wild type”

We have changed all instances of “wild-type” to “wild type”.

Line 204: Use qRT-PCR instead of RT-PCR

We have revised “RT-PCR” to “qRT-PCR”

Lines 253 ff.: The revertant strain ∆agr/pvlrev should be identical to the wild type, but Figure 5 shows less induction by C. albicans. Is there an explanation?

One explanation could be assay variability that may not necessarily be meaningful biologically, as there is only a 10% difference in cell death relative to co-culture for the parental *agr* mutant strain. As a precaution against a potential issue with the revertant strain, we repeated the experiments with a second independently generated clone of the *agr pvl^sbm^* mutant and the revertant strain for this clone. We observed similar levels of cytotoxicity induced by *Candida* co-culture for both the parental *agr* mutant and the *agr pvl^sbm^* revertant strain. We have included the data for this clone in place of the original figure (previously Figure 5, now Figure 4F).

Line 278: Since PVL is mainly found in sequence type 8 isolates, it would be informative, how big the percentage of ST8 isolates is compared to other STs. A non-PVL producing sequence type could have be used as a control for the experiments.

We thank the reviewer for this suggestion, and we have now included additional text in the discussion focusing on the frequency of PVL+ isolates, from lines 514-519. Data presented in Figure 5 do include a non-PVL producing strain, VUSA51, which is ST8 but was determined to be PVL-negative by whole genome sequencing.

Line 340: “a SaeR binding site”

This grammatical error has been corrected.

Line 507: Tris/HCl

This typo has been corrected.

**Reviewer #3:**

For the introduction I would have liked a few more words on the differences in susceptibility of murine and human cells to *S. aureus* and the factors involved (l. 105-115). As is now the reader is uncertain if the message is that the murine cells are representative enough or not.

We have updated the introduction (now lines 113-124) to include additional description and references that discuss the species tropism of *S. aureus* virulence factors.

Secondly from figure 2B and 3A it seems that Candida has the biggest impact on *S. aureus* cells that are *agr* mutants. However that could mimic *S. aureus* cells where the *agr* system is repressed for example by heterologous AIPs and so for *S. aureus* cells that may be considered to be in a "colonizing" state with repressed *agr* then the Candida can make a dramatic difference in terms of inducing cytotoxicity towards human monocytes. Interplay between *agr* and Sae could be commented on in the discussion

We thank the reviewer for this insightful comment and have included new text in the Discussion section that highlights several potential clinical scenarios where *C. albicans* may regulate the virulence of *S. aureus* strains with dysfunctional or inhibited *agr.* This text is in lines 473-488.
