# Supplementary material for: Candida albicans activates Staphylococcus aureus virulence regulatory systems to drive toxin-mediated human cell death
Source: PLoS Pathog. 2026 Aug 5;22(8):e1014490. doi: 10.1371/journal.ppat.1014490 (PMC13475977; doi:10.1371/journal.ppat.1014490)
Supplement: Peer review history

# Peer review: Decision Letter 0

**DOI:** 10.1371/journal.ppat.1014490.r001

**Authors:** Alice Prince, Michael Otto

25 Jan 2026

*Candida albicans*  activates *Staphylococcus aureus*  virulence regulatory systems to drive toxin-mediated human cell death

PLOS Pathogens

Dear Dr. Cassat,

All reviewers agreed that the presented experiments and text only need minor adjustments. However, reviewer 1 notes that the manuscript only marginally advances our understanding compared to previous publications, including particularly from your group. In reviewing the literature, I tend to agree with that assessment. For acceptance, you would need to provide additional data along the lines of what reviewer 1 notes: a clinically relevant animal model or description of the mechanism by which C. albicans enhances SaeRS activity that is different from the previously described mechanism for Agr.

Please submit your revised manuscript by Mar 26 2026 11:59PM. If you will need more time than this to complete your revisions, please reply to this message or contact the journal office at plospathogens@plos.org. When you're ready to submit your revision, log on to https://www.editorialmanager.com/ppathogens/ and select the 'Submissions Needing Revision' folder to locate your manuscript file.

Please include the following items when submitting your revised manuscript:

* A letter that responds to each point raised by the editor and reviewer(s). You should upload this letter as a separate file labeled 'Response to Reviewers'. This file does not need to include responses to any formatting updates and technical items listed in the 'Journal Requirements' section below.

* A marked-up copy of your manuscript that highlights changes made to the original version. You should upload this as a separate file labeled 'Revised Manuscript with Track Changes'.

* An unmarked version of your revised paper without tracked changes. You should upload this as a separate file labeled 'Manuscript'.

If you would like to make changes to your financial disclosure, competing interests statement, or data availability statement, please make these updates within the submission form at the time of resubmission. Guidelines for resubmitting your figure files are available below the reviewer comments at the end of this letter.

We look forward to receiving your revised manuscript.

Kind regards,

Michael Otto

Section Editor

PLOS Pathogens

Alice Prince

Section Editor

PLOS Pathogens

Sumita Bhaduri-McIntosh

Editor-in-Chief

PLOS Pathogens

orcid.org/0000-0003-2946-9497

Michael Malim

PLOS Pathogens

orcid.org/0000-0002-7699-2064

**Journal Requirements:**

If the reviewer comments include a recommendation to cite specific previously published works, please review and evaluate these publications to determine whether they are relevant and should be cited. There is no requirement to cite these works unless the editor has indicated otherwise.

1) Please ensure that the CRediT author contributions listed for every co-author are completed accurately and in full.

At this stage, the following Authors/Authors require contributions: Kara Eichelberger, Nicholas A Podar, Jia A Mei, Jacob M Curry, Ravishankar Chandrasekaran, Saikat Paul, Julie A Bastarache, Victor J Torres, Paul L Fidel Jr., Brian M Peters, and James E Cassat. Please ensure that the full contributions of each author are acknowledged in the "Add/Edit/Remove Authors" section of our submission form.

The list of CRediT author contributions may be found here: https://journals.plos.org/plospathogens/s/authorship#loc-author-contributions

2) We ask that a manuscript source file is provided at Revision. Please upload your manuscript file as a .doc, .docx, .rtf or .tex. If you are providing a .tex file, please upload it under the item type u2018LaTeX Source Fileu2019 and leave your .pdf version as the item type u2018Manuscriptu2019.

3) Please upload all main figures as separate Figure files in .tif or .eps format. For more information about how to convert and format your figure files please see our guidelines:

https://journals.plos.org/plospathogens/s/figures

4) We have noticed that you have uploaded Supporting Information files, but you have not included a list of legends. Please add a full list of legends for your Supporting Information files after the references list.

5) Please send a completed 'Competing Interests' statement, including any COIs declared by your co-authors. If you have no competing interests to declare, please state "The authors have declared that no competing interests exist". Otherwise please declare all competing interests beginning with the statement "I have read the journal's policy and the authors of this manuscript have the following competing interests"

**Reviewers' Comments:**

Reviewer's Responses to Questions

**Part I - Summary**

Please use this section to discuss strengths/weaknesses of study, novelty/significance, general execution and scholarship.

Reviewer #1: In this manuscript by Eichelberger et al., the authors build on a body of work demonstrating that the fungal pathogen Candida albicans can augment disease severity during co-infection with the bacterium Staphylococcus aureus. Previous work by the group demonstrated that fungal metabolite consumption increased S. aureus quorum sensing activity, leading to toxin expression and increased disease severity. In the current work, the authors describe increased toxin expression by S. aureus in co-culture with C. albicans that is independent of the Agr quorum-sensing system. The enhancement of S. aureus virulence in co-culture was observed to be Agr-dependent against murine cells, but significant Agr-independent activity remained against human immune cells. Promoter activity and qRT-PCR assays demonstrated that in the absence of the Agr system, co-culture increased activity of the SaeRS two-component system and subsequent Panton-Valentine leukocidin expression. Isogeneic deletion and complementation studies confirmed the role of PVL in human cell cytotoxicity and broad reproducibility of this phenotype was observed for a variety of similar sequence type strains of S. aureus. While this work is technically sound and clearly identifies PVL as the human-specific toxin that is induced upon C. albicans co-culture, this discovery does feel like a marginal improvement upon what has been previously described already, and clear clinical relevance is not described. Importantly, the paper does not address how C. albicans induces SaeRS activity in the absence of Agr and therefore only describes one half of the mechanism of this interaction.

Reviewer #2: The manuscript of Eichelberger et al describes a novel mechanism in the interaction between Candida albicans and Staphylococcus aureus. It is well known that the two species can be frequently isolated together from polymicrobial infections, and that C. albicans upregulates the virulence of S. aureus by induction of the staphylococcal Agr quorum sensing system. This could be confirmed by the work of Eichelberger et al. The production of alpha-type phenol-soluble modulins (PSM alpha), which are regulated by agr, seems to be the responsible mechanism by which S. aureus killed murine monocytes, since a psm alpha mutant revealed hardly any cytotoxicity. Nevertheless, the presence of C. albicans still induced cytotoxicity in the psm alpha mutant and the authors could identify gamma-hemolysin as responsible factor, indicating that two staphylococcal virulence factors together are responsible for cytotoxicity in murine monocytes.

Interestingly, analysis of human cell lines showed the inducing effect of C. albicans even on the agr-mutant of S. aureus, indicating that a human-specific toxin was upregulated even in the “avirulent” agr-deletion background. Whereas monocytes and neutrophils were sensitive, human epithelial and endothelial cell lines were not affected. Here, the authors could show that the expression of the factor is SaeRS dependent, a second well-known global regulator in S. aureus, and that the Panton-Valentine-leukocidin (PVL, encoded by lukSF-PV) is the toxin which affects human monocytes and neutrophils. This could be confirmed by inhibition of the PVL receptor, which led to strongly reduced cytotoxicity. Finally, the authors could show that the observed mechanism is a general mechanism since randomly selected clinical isolates of S. aureus and C. albicans all showed the same inducing effect, although at varying levels. Since PVL is a human specific factor, no animal models could be performed, but the results on human cell lines are very clear.

Reviewer #3: The strength of the manuscript is that it addresses and important and understudied topic namely co-infections of pathogens and how they affect each other. Here the interaction involves the impact of Candida albicans on S. aureus and it is shown to be mediated by the central agr regulatory locus as well as by the SaeRS system that in the absence of agr is stimulating the cytotoxicicity by Candida by driving the expression of the PVL toxin. Experiments appear carefully conducted and well controlled and the results are communicated in an easy to follow manner.

**********

**Part II – Major Issues: Key Experiments Required for Acceptance**

Please use this section to detail the key new experiments or modifications of existing experiments that should be absolutely required to validate study conclusions.

Generally, there should be no more than 3 such required experiments or major modifications for a "Major Revision" recommendation. If more than 3 experiments are necessary to validate the study conclusions, then you are encouraged to recommend "Reject".

Reviewer #1: The group’s 2024 paper (https://doi.org/10.1038/s41467-024-50058-w) shows that ribose consumption by C. albicans activates Agr in S. aureus. This is an easy experiment to demonstrate that the phenotype observed with SaeRS is independent of the previously described ribose repression.

It appears that the enhancement of virulence via the Agr system has a larger functional effect (PSMs, Hlg, PVL all regulated by Agr). Therefore, the SaeRS mediated mechanism is only relevant in situations where the Agr system is deficient or not activated. The authors have not made a clear and compelling case regarding the clinical relevance of this scenario. While there are some niches that encourage selection of Agr mutations, such as implant associated biofilm infections, is there any evidence to suggest this regularly occurs in polymicrobial infections, such as chronic wounds?

Unlike in other figures, in Figure 7, there are no controls to demonstrate that the degree of S. aureus cytotoxicity enhancement (or lack of) is not due to differences in S. aureus growth.

Reviewer #2: (No Response)

Reviewer #3: There are no major issues with this manuscript. The experiments appear very well conducted and the results are very well and clearly presented. Overall, the manuscript is very convincing.

**********

**Part III – Minor Issues: Editorial and Data Presentation Modifications**

Please use this section for editorial suggestions as well as relatively minor modifications of existing data that would enhance clarity.

Reviewer #1: In Figure S3 the caption states that samples taken every 30 minutes but the graph appears to only show data points each hour.

Line 194: “These data support that both Agr and SaeRS-regulated toxins are required for human cell death.” I disagree with the phrasing of this sentence. The data in Figure 3a show that C. albicans can enhance toxicity through either SaeRS or Agr. Furthermore, PVL is known to be regulated by both SaeRS and Agr, so signaling through either system could enhance PVL cytotoxicity. These data also do not distinguish whether PVL is the only toxin that is sufficient for human cell death or if Agr-regulated α-PSMs also will cause human cell death.

In Figure 7, I would think that the more important statistical test for these two figures would be to compare the clinical strains to the mono-culture to assess whether all clinical strains can significantly increase S. aureus cytotoxicity. As it is, the statistical analysis appears to ask whether each strain induces more or less cytotoxicity than the reference strain, which is a slightly different question than what is framed in the paper (Line 291- “These data indicate that the ability to enhance S. aureus cytotoxicity is broadly conserved among C. albicans isolates…”).

Reviewer #2: This manuscript is well-written and easy to understand. There are only some minor comments:

The authors use “wild-type” in the entire manuscript, which is an adjective and should be replaced by “wild type”

Line 204: Use qRT-PCR instead of RT-PCR

Lines 253 ff.: The revertant strain ∆agr/pvlrev should be identical to the wild type, but Figure 5 shows less induction by C. albicans. Is there an explanation?

Line 278: Since PVL is mainly found in sequence type 8 isolates, it would be informative, how big the percentage of ST8 isolates is compared to other STs. A non-PVL producing sequence type could have be used as a control for the experiments.

Line 340: “a SaeR binding site”

Line 507: Tris/HCl

Reviewer #3: For the introduction I would have liked a few more words on the differences in susceptibility of murine and human cells to S. aureus and the factors involved (l. 105-115). As is now the reader is uncertain if the message is that the murine cells are representative enough or not.

Secondly from figure 2B and 3A it seems that Candida has the biggest impact on S. aureus cells that are agr mutants. However that could mimick S. aureus cells where the agr system is repressed for example by heterologous AIPs and so for S. aureus cells that may be considered to be in a "colonizing" state with repressed agr then the Candida can make a dramatic difference in terms of inducing cytotoxicity towards human monocytes. Interplay between agr and Sae could be commented on in the discussion.

**********

PLOS authors have the option to publish the peer review history of their article (what does this mean?). If published, this will include your full peer review and any attached files.

If you choose “no”, your identity will remain anonymous but your review may still be made public.

**Do you want your identity to be public for this peer review?**  For information about this choice, including consent withdrawal, please see our Privacy Policy.

Reviewer #1: No

Reviewer #2: No

Reviewer #3: No

**Figure resubmission:**

**Reproducibility:**

?>

# Author comment: Author response to Decision Letter 1

**DOI:** 10.1371/journal.ppat.1014490.r002

14 Jul 2026

Attachment Submitted filename: Eichelberger et al - response to reviewers.docx

# Editor report: Decision Letter 1

**DOI:** 10.1371/journal.ppat.1014490.r003

**Authors:** Alice Prince, Michael Otto

21 Jul 2026

Dear Dr. Cassat,

We are pleased to inform you that your manuscript '*Candida albicans*  activates *Staphylococcus aureus*  virulence regulatory systems to drive toxin-mediated human cell death' has been provisionally accepted for publication in PLOS Pathogens.

Before your manuscript can be formally accepted you will need to complete some formatting changes, which you will receive in a follow up email. A member of our team will be in touch with a set of requests.

Please note that your manuscript will not be scheduled for publication until you have made the required changes, so a swift response is appreciated.

IMPORTANT: The editorial review process is now complete. PLOS will only permit corrections to spelling, formatting or significant scientific errors from this point onwards. Requests for major changes, or any which affect the scientific understanding of your work, will cause delays to the publication date of your manuscript.

Should you, your institution's press office or the journal office choose to press release your paper, you will automatically be opted out of early publication. We ask that you notify us now if you or your institution is planning to press release the article. All press must be co-ordinated with PLOS.

Thank you again for supporting Open Access publishing; we are looking forward to publishing your work in PLOS Pathogens.

Best regards,

Michael Otto

Section Editor

PLOS Pathogens

Alice Prince

Section Editor

PLOS Pathogens

Sumita Bhaduri-McIntosh

Editor-in-Chief

PLOS Pathogens

orcid.org/0000-0003-2946-9497

Michael Malim

Editor-in-Chief

PLOS Pathogens

orcid.org/0000-0002-7699-2064

***********************************************************

Reviewer Comments (if any, and for reference):

# Editor report: Acceptance letter

**DOI:** 10.1371/journal.ppat.1014490.r004

**Authors:** Alice Prince, Michael Otto

Dear Dr. Cassat,

We are delighted to inform you that your manuscript, "*Candida albicans*  activates *Staphylococcus aureus*  virulence regulatory systems to drive toxin-mediated human cell death," has been formally accepted for publication in PLOS Pathogens.

We have now passed your article onto the PLOS Production Department who will complete the rest of the pre-publication process. All authors will receive a confirmation email upon publication.

The corresponding author will soon be receiving a typeset proof for review, to ensure errors have not been introduced during production. Please review the PDF proof of your manuscript carefully, as this is the last chance to correct any scientific or type-setting errors. Please note that major changes, or those which affect the scientific understanding of the work, will likely cause delays to the publication date of your manuscript. Note: Proofs for Front Matter articles (Pearls, Reviews, Opinions, etc...) are generated on a different schedule and may not be made available as quickly.

Soon after your final files are uploaded, the early version of your manuscript, if you opted to have an early version of your article, will be published online. The date of the early version will be your article's publication date. The final article will be published to the same URL, and all versions of the paper will be accessible to readers.

For Research Articles, you will receive an invoice from PLOS for your publication fee after your manuscript has reached the completed accept phase. If you receive an email requesting payment before acceptance or for any other service, this may be a phishing scheme. Learn how to identify phishing emails and protect your accounts at https://explore.plos.org/phishing.

Thank you again for supporting open-access publishing; we are looking forward to publishing your work in PLOS Pathogens.

Best regards,

Sumita Bhaduri-McIntosh

Editor-in-Chief

PLOS Pathogens

orcid.org/0000-0003-2946-9497

Michael Malim

Editor-in-Chief

PLOS Pathogens

orcid.org/0000-0002-7699-2064
